# Mitochondrial transfer in the tumor microenvironment: a dynamic determinant of cancer plasticity, immune dysfunction, and therapeutic opportunity

**DOI:** 10.3389/fimmu.2026.1871148

**Published:** 2026-06-03

**Authors:** Zhenyuan Xu, Qingli Zhou, Jifei Miao

**Affiliations:** Shenzhen Baoan Traditional Chinese Medicine Hospital, Guangzhou University of Chinese Medicine, Guang Dong, China

**Keywords:** cancer plasticity, extracellular vesicles, mitochondrial transfer, t cell exhaustion, tumor microenvironment, tunneling nanotubes

## Abstract

Intercellular mitochondrial transfer has emerged as a significant mode of communication within the tumor microenvironment (TME). We propose that this process operates as a stress-adaptive organelle economy, redistributing three biologically decisive assets (respiratory competence, redox tolerance, and stress history) among tumor, immune, and stromal cells according to local metabolic asymmetry. Cancer cells acquire healthy mitochondria from stromal and immune populations, thereby restoring oxidative phosphorylation, expanding metabolic plasticity, and driving chemoresistance. Tumor cells also engage in outward transfer that is recipient-selective. Damaged mitochondria may be exported to CD8^+^ T cells and fibroblasts, corrupting effector function and reprogramming the stroma, whereas functional mitochondria may be delivered to pro-tumor immune populations such as M2 tumor-associated macrophages, myeloid-derived suppressor cells, and regulatory T cells to sustain their immunosuppressive activity. Functional mitochondria therefore play a dual role in tumorigenesis. The consequences for antitumor immunity depend on donor identity, cargo quality, and recipient lineage rather than on transfer itself. The principal transport routes are tunneling nanotubes, extracellular vesicles, and cell fusion, but biological outcome is ultimately governed by a post-transfer fate checkpoint involving PINK1/Parkin-mediated mitophagy and USP30-facilitated retention. Therapeutically, the goal is not to block or enhance transfer globally but to achieve context-selective modulation within an inherently bidirectional system.

## Graphical Abstract

**Figure:**
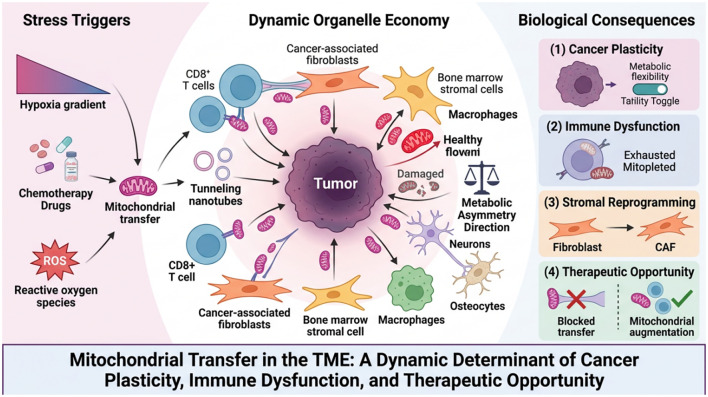
Mitochondrial transfer in the tumor microenvironment operates as a dynamic organelle economy. Stress triggers — hypoxia, ROS, and chemotherapy — drive bidirectional exchange between tumor cells and surrounding immune, stromal, and mesenchymal populations via tunneling nanotubes and extracellular vesicles. Metabolic asymmetry dictates transfer directionality: tumors acquire healthy mitochondria while offloading damaged organelles. Four major biological consequences emerge: enhanced cancer plasticity, immune dysfunction through T cell mitochondrial depletion, stromal reprogramming toward cancer-associated fibroblast phenotypes, and context-dependent therapeutic opportunities through selective blockade or mitochondrial augmentation strategies.

## 1 Introduction

Mitochondria influence tumor biology through ATP production, redox control, biosynthesis, calcium handling, apoptosis, innate immune signaling, and metabolite-dependent regulation of transcriptional and epigenetic state (1, 2). These functions are often discussed as cell-intrinsic properties. However, accumulating evidence indicates that mitochondria, mtDNA, and mitochondria-derived cargo can move between tumor cells and neighboring stromal or immune cells, thereby altering the metabolic and functional state of both donor and recipient populations (3, 4).

This intercellular dimension is important because many features of tumor progression are difficult to explain using purely cell-autonomous models. Tumor cells exposed to hypoxia, nutrient deprivation, chemotherapy, radiotherapy, or immune pressure often recover respiratory function or stress tolerance despite mitochondrial injury. In several systems, this recovery is supported by acquisition of mitochondria or mtDNA from stromal, endothelial, immune, or tissue-resident cells (5, 6). Tumor cells can also export mitochondrial material to immune and stromal compartments, where it may impair cytotoxic lymphocytes, reprogram fibroblasts, or support immunosuppressive cell populations (7, 8). Thus, mitochondrial transfer is not simply a rescue mechanism for damaged cells; it is a bidirectional process that can redistribute metabolic capacity and mitochondrial burden within the TME. A more defensible interpretation treats it as a conditional mode of communication that becomes biologically meaningful when donor and recipient differ sharply in mitochondrial fitness, when stress enhances physical connectivity, and when transferred organelles evade degradation.

The TME is precisely such a setting. Tumor cells, fibroblasts, endothelial cells, macrophages, platelets, neurons, MSCs, and lymphocytes coexist under sustained metabolic and inflammatory pressure (9, 10), with markedly different mitochondrial dependencies (10–12). The TME is therefore not only a place where cells compete for metabolites, but one where they can exchange the organelle machinery that determines how metabolites are used.

Several excellent studies have catalogued the routes by which transfer occurs, including tunneling nanotubes (TNTs), extracellular vesicles (EVs), cell fusion, and uptake of cell-free mitochondria or mitochondrial fragments (13–15). Yet what is still lacking in many reviews is a coherent conceptual framework that explains why mitochondrial transfer matters beyond descriptive novelty. We argue that the key contribution of this process is not merely “metabolic rescue.” Rather, mitochondrial transfer redistributes three biologically decisive assets within the TME: respiratory competence, redox tolerance, and stress history. Functional mitochondria can restore oxidative phosphorylation in damaged recipient cells; dysfunctional mitochondria can export oxidative burden; mtDNA can rescue electron transport, and—although direct evidence in the context of intercellular transfer remains limited—has been hypothesized to contribute to genomic instability through numtogenesis under certain conditions (16–18). The outcome depends on who donates, who receives, what is transferred, and what happens to the incoming material after it arrives.

This distinction directly affects how the field interprets transfer between cancer cells and T cells, between stromal cells and leukemia, between macrophages and breast cancer cells, or between neurons and metastatic tumor cells (19–21). In some settings, transfer promotes tumor growth by restoring respiration. In others, it undermines immunity by depriving T cells of mitochondria or burdening them with tumor-derived organelles. In still others, it may support tissue repair or even activate antitumor pathways, as suggested by osteocyte-mediated transfer in bone metastasis (22). A mature view of the field must therefore move beyond the assumption that mitochondrial transfer is intrinsically pro-tumorigenic or intrinsically reparative. It is neither. It is context-governed.

Another reason this topic deserves careful treatment is that mitochondrial transfer is beginning to move toward the clinic. Artificial mitochondrial transfer and mitochondrial transplantation are being explored in mitochondrial disease, ischemic injury, inflammatory disorders, and regenerative medicine (23–26). In parallel, the oncology field is considering how to block transfer routes that support chemoresistance or immune evasion (27, 28). But translational enthusiasm can become premature if it is not grounded in mechanistic discrimination. A strategy that globally suppresses organelle exchange may impair antitumor immunity or tissue recovery. Conversely, therapeutic mitochondrial augmentation could unintentionally benefit tumor cells if not spatially or cellularly restricted. The therapeutic problem, then, is not simply whether to inhibit or enhance transfer. It is how to control directionality and recipient specificity.

## 2 Mitochondrial fitness as a limiting variable in tumors and immune cells

This section explains why even limited mitochondrial transfer can have large biological effects in tumors, particularly when recipient cells are already constrained by respiratory stress, redox imbalance, or impaired mitochondrial quality control.

### 2.1 Tumor-cell mitochondrial fitness

Understanding mitochondrial transfer first requires defining why mitochondrial fitness limits tumor-cell behavior within the TME. Mitochondria regulate not only ATP production through oxidative phosphorylation (OXPHOS), but also ROS generation, calcium signaling, apoptosis, and metabolite-dependent biosynthetic and epigenetic programs (1, 2). In cancer, these functions are unusually consequential because tumor cells live under chronic selective pressure. A small change in mitochondrial competence can alter whether a cell survives oxidative stress, persists under nutrient shortage, enters apoptosis, or acquires metastatic potential.

This is one reason the traditional caricature of cancer metabolism as “glycolytic rather than mitochondrial” has become untenable. The Warburg effect remains biologically important: many tumor cells favor glycolysis even in the presence of oxygen, and that shift supports biosynthesis, redox adaptation, and immunosuppressive niche formation (9, 29). But the field has moved beyond the mistaken implication that glycolysis renders mitochondria dispensable. In reality, many tumors retain substantial dependence on mitochondrial respiration, especially during metastatic dissemination, stem-like maintenance, recovery from treatment, and adaptation to fluctuating oxygen or nutrient availability (19, 30). What tumors often gain is not freedom from mitochondria, but flexibility in how mitochondrial and glycolytic programs are balanced.

That flexibility is partly constrained by mitochondrial integrity. mtDNA is highly susceptible to damage because it lies close to the electron transport chain, lacks histone protection, and is supported by relatively limited repair mechanisms (31). As a result, mtDNA mutations and copy number alterations are common in cancer and can influence metabolism, ROS generation, treatment response, and metastatic behavior (32, 33). Nuclear-encoded mitochondrial enzymes are also frequent targets of oncogenic disruption. Mutations in IDH1/2, SDH, FH, and TRAP1 alter the mitochondrial metabolite landscape and generate oncometabolites capable of reshaping chromatin and transcription (32). Yet the literature is not entirely linear on this point. Some mtDNA mutations appear to promote metastasis, whereas others may suppress dissemination or trigger apoptosis (34, 35). Rather than treating these findings as contradictions, it is more useful to recognize that mitochondrial dysfunction is not a single state. The biological effect depends on whether the altered mitochondrial program remains compatible with stress survival.

### 2.2 Immune-cell mitochondrial requirements

This complexity becomes even more pronounced in the TME. Tumor cells are surrounded by cells with distinct metabolic identities and distinct mitochondrial dependencies. T cells are a prime example. Early activation depends heavily on glycolysis, but sustained effector function, cytokine production, memory differentiation, and long-term persistence all require preserved mitochondrial fitness (12, 36). Exhausted tumor-infiltrating lymphocytes show reduced mitochondrial biogenesis, lower respiratory reserve, elevated oxidative stress, and suppression of PGC-1α-driven programs (37, 38). PD-1 signaling aggravates this condition by inhibiting both glycolytic and mitochondrial metabolism (37). Once this state becomes entrenched, T cells do not merely become less energetic; they lose the metabolic architecture needed to maintain antitumor function.

Other immune populations are equally shaped by mitochondrial state, although in different ways. Regulatory T cells and myeloid-derived suppressor cells often rely on high mitochondrial activity to support suppressive functions (10). Tumor-associated macrophages (TAMs) adopt metabolic programs involving fatty acid oxidation and OXPHOS that align with tissue remodeling, angiogenesis, and immunosuppression (39, 40). Dendritic cells switch toward glycolysis during activation but can be functionally impaired by oxidative stress (10). Thus, when mitochondrial transfer occurs in the TME, it is not entering a metabolically neutral environment. It is intervening in a pre-existing set of lineage-specific dependencies.

### 2.3 Mitochondrial fitness as a community-level variable

A recipient cell is not just receiving extra organelles. It may be receiving the capacity to restore respiration, dampen ROS, expand biosynthetic range, resist apoptosis, or alter differentiation. Conversely, a donor cell may be losing not merely a fraction of its mitochondrial mass, but a component of its own stress-buffering reserve. This point has been underappreciated in some descriptions of mitochondrial exchange (41). Because most cells contain many mitochondria, one might assume that transfer of a limited number of organelles is biologically trivial. In systems where mitochondrial function is already limiting, small shifts can have disproportionate effects, especially if the incoming organelles are selectively retained or if donor cells are pushed past a functional threshold.

We propose that mitochondrial status should therefore be treated as a community-level variable in tumors rather than a purely intracellular one. The TME contains cells with different mitochondrial capacities, and transfer allows those capacities to be redistributed. A stromal cell with preserved respiration may become a donor reservoir. A tumor cell with mtDNA damage may become a recipient. A T cell under chronic antigenic stress may become vulnerable not only because its own mitochondria fail, but because tumor cells can actively drain or overwrite its mitochondrial pool (7, 42). Once viewed this way, mitochondrial transfer is not an isolated phenomenon layered on top of tumor metabolism. It is one of the mechanisms by which metabolic inequality between neighboring cells is translated into functional asymmetry.

## 3 Conceptual framework: directionality, cargo quality, and post-transfer fate

Having established why mitochondrial fitness matters, the next step is to define which transfer events are biologically meaningful and which variables determine their outcomes.

### 3.1 What counts as biologically meaningful transfer?

The term “mitochondrial transfer” includes several biologically distinct processes that should not be treated as equivalent. It sounds singular, as though one well-defined biological event has been observed across systems. In reality, the field encompasses a spectrum of processes: transfer of intact mitochondria, transfer of mtDNA, release of mitochondrial fragments, EV-mediated trafficking of mitochondrial components, and, in some contexts, transfer followed by stable replacement of endogenous mitochondrial populations (13, 14, 43, 44). Lumping all of these under one label can create conceptual imprecision. In this Review, we define biologically meaningful mitochondrial transfer as donor-derived mitochondrial material entering a recipient cell and producing a measurable change in recipient metabolism, signaling, survival, differentiation, immune function, or treatment response. The first task, therefore, is to decide what biological logic unites these observations.

### 3.2 Directionality and metabolic asymmetry

We propose that many transfer events can be understood as stress-adaptive redistribution of mitochondrial function or mitochondrial burden. In this model, mitochondrial transfer acts as a means of reallocating organelle function and organelle burden across cells living under uneven stress. Cells with severe respiratory insufficiency, mitochondrial DNA loss, or therapy-induced damage tend to seek compensatory inputs. Cells with greater reserve, or simply with more advantageous structural positioning, can become donors. At the same time, cells can export defective mitochondria to relieve their own burden. Transfer direction is therefore not fixed. It is an emergent property of local metabolic asymmetry.

Before developing this framework further, we wish to be explicit about its epistemic status. The notions of an “organelle economy” and “stress-adaptive redistribution” are proposed here as interpretive frameworks, not as experimentally validated mechanisms. The individual components on which they rest are supported by direct evidence: stromal-to-tumor mitochondrial donation in AML and solid tumors (20, 45, 46), tumor-to-T-cell transfer of mutant mitochondria (7, 42), CAF induction through tumor-derived mitochondria (8), and osteocyte-mediated cGAS/STING activation in bone metastasis (22). What remains hypothesis is the integrative claim that these disparate observations constitute a single adaptive system whose directionality is governed by local bioenergetic asymmetry. We adopt this framing because it organizes otherwise contradictory findings into a tractable conceptual structure, but we note that formal proof would require longitudinal, fitness-resolved measurements of donor and recipient cells before, during, and after transfer—data that the field does not yet possess.

This framework helps explain why donor-recipient roles vary across reports. In leukemia, stromal cells often supply mitochondria to malignant cells, restoring OXPHOS and survival under chemotherapy (20, 45). In breast and lung cancer models, tumor cells can capture mitochondria from immune cells, weakening lymphocyte function while increasing their own fitness (47). In other contexts, tumor cells themselves become donors, exporting damaged or mutant mitochondria to T cells or fibroblasts, thereby impairing immunity or reprogramming stromal state (8). They reflect different positions within the same adaptive network.

A second point follows from this. The direction of transfer is likely determined less by cell identity per se than by relative mitochondrial fitness at a given moment. It is tempting to categorize certain cell types as “natural donors” and others as “natural recipients,” but the evidence does not support a rigid taxonomy. The same tumor cell may be a recipient when its respiratory function is impaired and a donor when it needs to unload damaged organelles. The same stromal compartment may support immunity in one context yet facilitate tumor resilience in another. This dynamic view fits better with the TME as an environment characterized by fluctuating hypoxia, inflammatory cues, and therapy-induced injury (48, 49).

### 3.3 Cargo quality and recipient selection

Much of the literature focuses on transport routes, especially TNTs, but transport alone does not determine biological effect. What matters is whether the incoming mitochondrial material persists, integrates, or is degraded. This is where the concept of quality control becomes central. A transferred mitochondrion that is immediately recognized as damaged and eliminated by mitophagy is biologically different from one that fuses into the host network, replicates, and is inherited by daughter cells (50, 51). The work showing that tumor-derived mitochondria can carry USP30 into T cells is especially illuminating in this regard. It suggests that transfer can reshape the intracellular balance of mitophagic selection, allowing incoming organelles to survive while endogenous host mitochondria are removed (7). That is a qualitatively different process from transient uptake.

From this perspective, the key variables in any mitochondrial transfer event are not only donor, recipient, and route, but also cargo quality and post-transfer fate. This may explain why the literature sometimes produces divergent outcomes even when similar cell types are involved. Healthy mitochondria from stromal donors may restore function in one recipient, whereas ROS-rich or depolarized mitochondria from inflammatory donors may trigger stress pathways in another (21, 52). Likewise, transfer of mtDNA can rescue respiration in ρ0 cells (5, 6). Whether chronic mitochondrial stress associated with intercellular transfer also promotes mtDNA release and subsequent nuclear integration—thereby increasing NUMT burden and potentially affecting genome stability—remains a speculative possibility rather than an established consequence, as direct mechanistic evidence linking transfer events to numtogenesis is currently lacking (16, 17). If the field is to mature, it will need to stop asking simply whether transfer happened and ask instead what sort of mitochondrial material moved, under what selective conditions, and with what intracellular consequences.

### 3.4 Ecological interpretation of mitochondrial exchange

**Figure 1:**
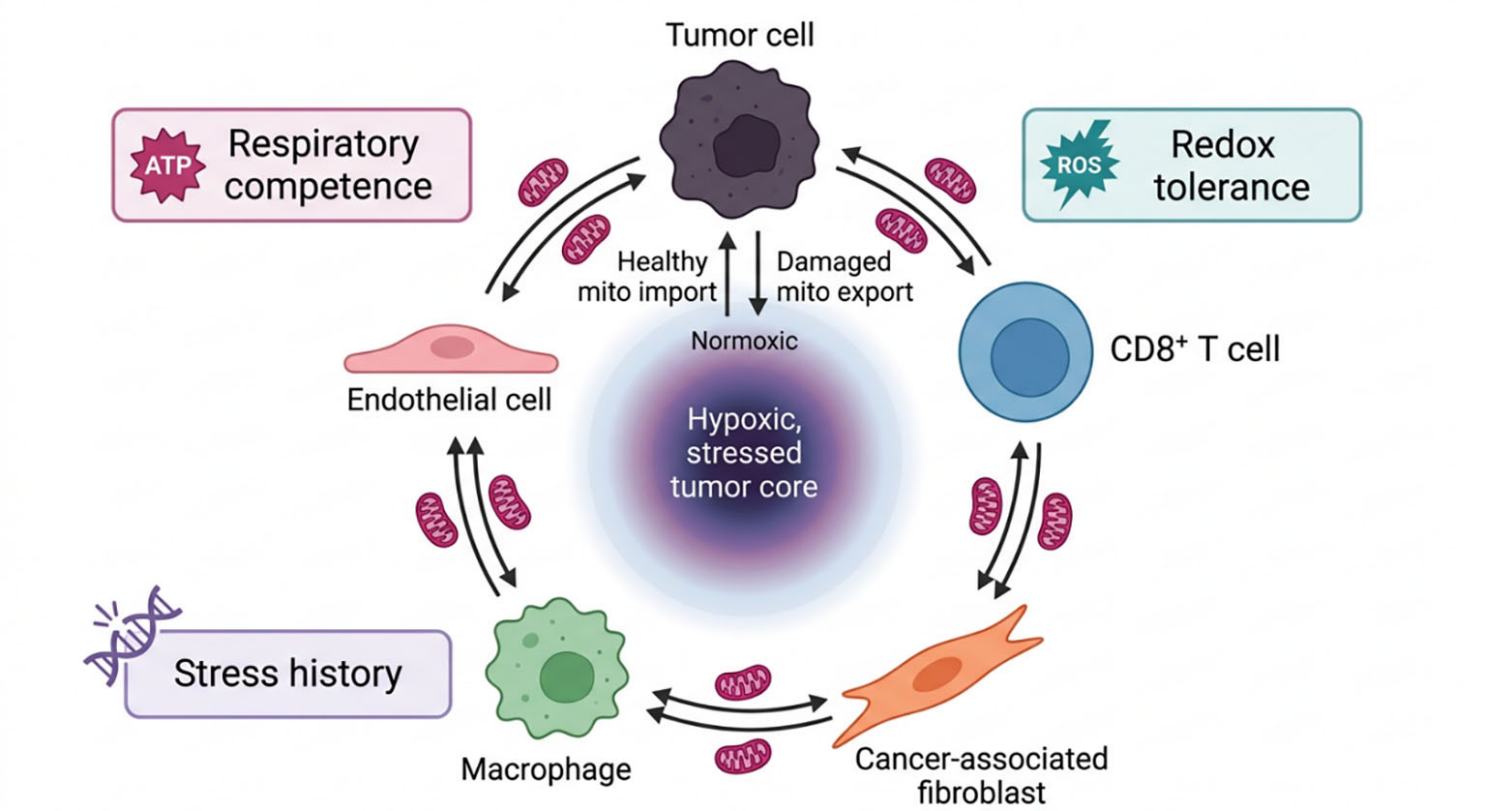
The organelle economy: stress-driven mitochondrial redistribution in the TME. Within the TME, metabolic stress—particularly hypoxia—drives bidirectional mitochondrial exchange among tumor cells, CD8^+^ T cells, cancer-associated fibroblasts, macrophages, and endothelial cells. Tumor cells import healthy mitochondria from neighboring cells while exporting damaged organelles, thereby redistributing three biologically decisive assets: respiratory competence (ATP-generating capacity), redox tolerance (ROS buffering), and stress history (mtDNA integrity). Transfer direction is not fixed but emerges from local bioenergetic asymmetry, establishing a dynamic organelle economy that shapes cancer plasticity, immune function, and stromal reprogramming.

Ecological systems are defined by unequal access to resources, burden sharing, niche construction, and adaptation under stress. Functional mitochondria can be treated as scarce resources; damaged mitochondria can be treated as burdens; stromal reprogramming can be treated as niche construction; T cell impairment can be treated as competitive suppression. This ecological framing is not just rhetorical. It helps integrate cancer cell plasticity, immune exhaustion, and stromal activation into one model rather than describing them as unrelated consequences (**Figure 1**).

The most productive future studies, in our opinion, will be those that examine transfer as a competitive process with selective retention, rather than as a static event of cargo exchange. Such a perspective should also make it easier to reconcile the field’s apparent inconsistencies. Mitochondrial transfer is not uniformly protumorigenic, uniformly reparative, or uniformly deleterious. It is a conditional adaptation mechanism whose effects emerge from asymmetry—of mitochondrial quality, of stress intensity, and of the recipient’s ability to retain what it receives.

## 4 Cancer cells as recipients: restoring fitness, expanding plasticity

We first consider the best-supported direction of mitochondrial transfer in cancer: acquisition of mitochondria or mtDNA by malignant cells from non-malignant neighbors.

### 4.1 OXPHOS and mtDNA rescue

The strongest body of evidence in the field concerns acquisition of mitochondria or mtDNA by cancer cells from non-malignant neighbors, particularly under conditions of metabolic stress or therapy-induced injury. Tumor cells frequently operate near the limits of metabolic tolerance. They often carry mitochondrial defects, experience oxidative and nutrient stress, and are repeatedly injured by therapy. In that context, access to exogenous mitochondrial material can be highly advantageous.

The earliest proof of principle came from studies showing that mtDNA-deficient ρ0 tumor cells could regain respiration and proliferative capacity when co-cultured with stromal donors containing intact mtDNA (53). Since then, similar rescue phenomena have been documented across multiple tumor models (5, 6). The importance of these experiments is not simply historical. They established that mitochondrial defects in tumor cells are not necessarily terminal if neighboring cells can provide missing respiratory components. This finding should have permanently altered how the field interprets mitochondrial dysfunction in cancer. A damaged mitochondrial genotype within a tumor cell does not always imply an irreversibly damaged phenotype.

### 4.2 Metabolic plasticity, stemness, and metastasis

One obvious benefit of organelle acquisition is restoration of OXPHOS. Functional mitochondria can increase ATP production and improve the redox economy of recipient tumor cells (20, 28, 54). However, restoration of OXPHOS is important not only as an energy-producing process but also because it expands the metabolic options available to tumor cells. Restored OXPHOS allows tumor cells to survive fluctuating oxygen tension, manage ROS, support anabolic flux, and shift between proliferative, invasive, and stem-like states. In this sense, mitochondrial acquisition supports plasticity more than energy alone. In this sense, mitochondrial acquisition supports plasticity more than it supports energy alone.

The studies on cancer-associated fibroblasts (CAFs) make this particularly clear. CAF-derived mitochondria do not simply add more ATP to recipient cancer cells; they cooperate with lactate shuttling to activate the PGC-1α/SIRT1 axis and promote mitochondrial biogenesis and enhanced oxidative metabolism (46). In this setting, transfer amplifies an entire metabolic program. The resulting increase in mitochondrial ROS can activate Src/PKM2/HIF1 signaling, linking respiration to epithelial-mesenchymal transition (EMT), invasion, and metastatic competence (46). This sequence is revealing because it challenges the common intuition that reduced ROS is always the main benefit of healthier mitochondria. In some cases, a controlled rise in ROS becomes a signaling asset that promotes malignant progression.

Tumor stemness can be interpreted within the same framework because stem-like tumor cells often require flexible mitochondrial metabolism to survive stress, enter reversible quiescence, and reinitiate growth. Stem-like cancer cells do not thrive because they are permanently committed to one metabolic state. They thrive because they can shift states as conditions change. Neuron-to-cancer mitochondrial transfer, for example, has been linked to greater stemness and enhanced tolerance to metastatic stress (19). Glioblastoma cells receiving mitochondria from astrocytes show increased self-renewal and tumorigenicity (55). Such findings imply that imported mitochondria may be particularly valuable during moments of transition—dissemination, seeding, treatment escape—when metabolic rigidity becomes a liability.

### 4.3 Therapy resistance

A major functional consequence of mitochondrial acquisition is therapy resistance. This area has arguably provided the most clinically compelling evidence that mitochondrial transfer is not an incidental phenomenon. In AML, stromal donation of mitochondria sustains ATP production and oxidative metabolism, thereby reducing the cytotoxic impact of agents such as cytarabine and daunorubicin (20, 45). Similar themes have emerged in breast cancer, ovarian cancer, gastric cancer, multiple myeloma, and glioblastoma (27, 56). The mechanistic details vary, but the broader principle is consistent: treatments that rely, directly or indirectly, on mitochondrial damage, redox imbalance, or apoptosis induction can be blunted if recipient tumor cells replenish critical mitochondrial functions from neighboring donors.

However, the benefit of mitochondrial transfer extends beyond ATP supply. Several studies indicate that transfer can also stabilize membrane potential, reduce lethal ROS accumulation, suppress mitophagy, and alter cell death thresholds (57). In some cases, transferred mitochondria may function less as energy units and more as signaling nodes. The macrophage-to-cancer transfer described in breast cancer is a case in point: donated mitochondria persisted as a distinct population and generated localized ROS that promoted proliferation without necessarily integrating into the host mitochondrial network (21). This kind of signaling-centered transfer is conceptually important because it means that even a small organelle population, if spatially or biochemically privileged, can exert outsized effects.

**Figure 2:**
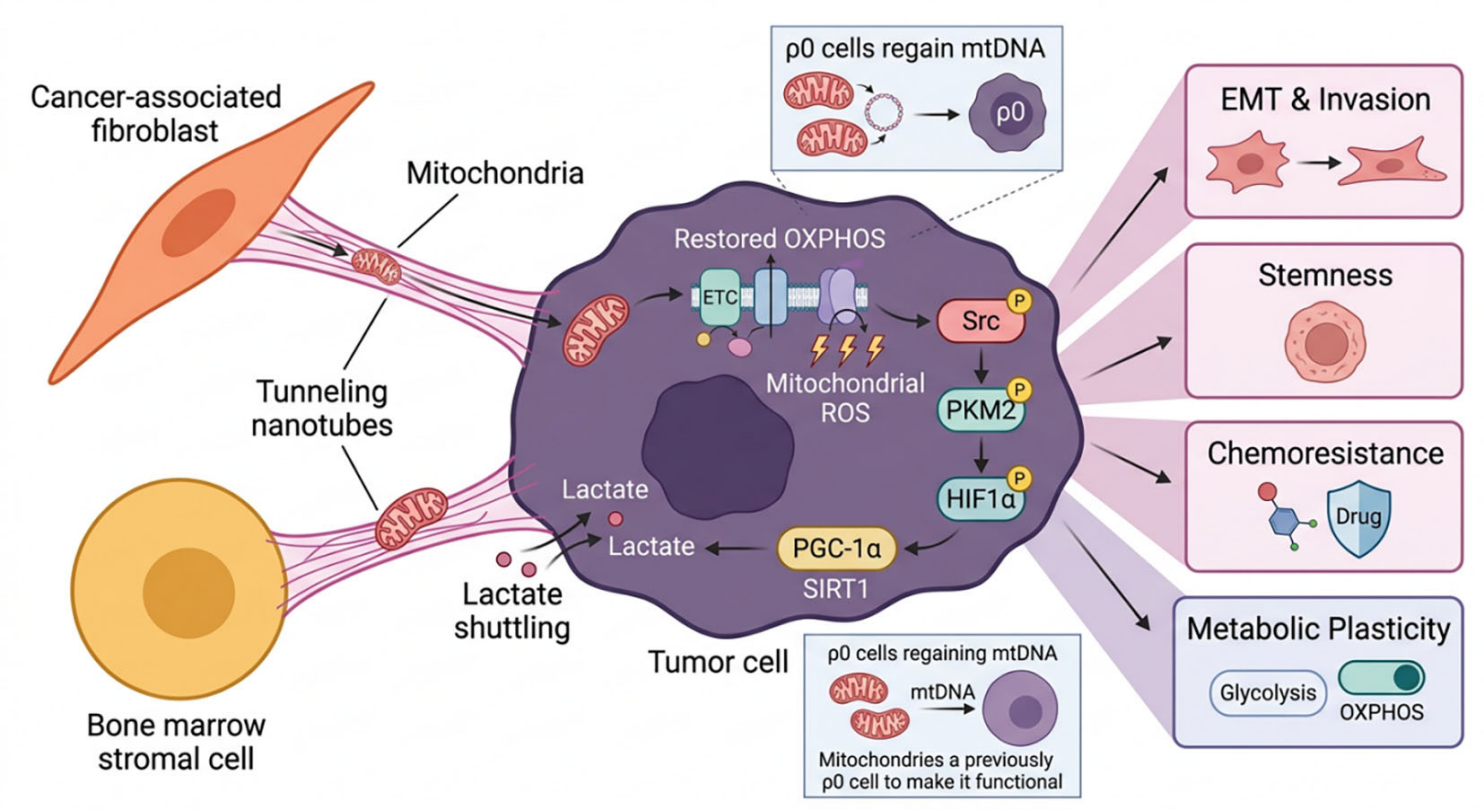
Mitochondrial acquisition fuels cancer cell plasticity. Cancer-associated fibroblasts (CAFs) and bone marrow stromal cells donate mitochondria to tumor cells via tunneling nanotubes. CAF-derived lactate cooperates with transferred mitochondria to activate the PGC-1α/SIRT1 axis, restoring OXPHOS and generating controlled mitochondrial ROS that drives Src/PKM2/HIF1α signaling. This cascade promotes four key phenotypic outcomes: EMT and invasion, stemness, chemoresistance, and metabolic plasticity (glycolysis–OXPHOS flexibility). Insets illustrate how mtDNA-deficient ρ0 tumor cells regain respiratory competence through acquisition of donor mtDNA, demonstrating that mitochondrial defects in cancer are not necessarily irreversible when stromal donors are available.

One of the most under-discussed consequences of mitochondrial transfer to tumor cells is the homogenization of stress tolerance across heterogeneous cancer populations. Tumors are metabolically diverse. Some subclones are highly respiratory, others strongly glycolytic; some are mitochondrially damaged, others relatively intact. If mitochondrial exchange occurs among tumor cells or from stromal donors to specific malignant subsets, then it can spread adaptive traits laterally rather than waiting for clonal selection to do the same job (58, 59). In practical terms, this means that mitochondrial transfer may accelerate the emergence of resistant or metastatic phenotypes by sharing metabolic competence between cells (**Figure 2**).

### 4.4 Evidence gaps and required controls

Despite these findings, several evidence gaps remain. Most evidence for tumor-benefiting transfer comes from experimental systems in which donor and recipient populations are relatively easy to distinguish. Demonstrating the same process rigorously in patient samples remains difficult (28). Moreover, the field likely overgeneralizes from high-visibility models such as AML or glioblastoma. It is not yet clear whether mitochondrial acquisition is equally important across all solid tumors, or whether certain tissue architectures, donor abundances, or treatment pressures are particularly permissive. A more discriminating literature would ask not simply whether cancer cells can receive mitochondria, but under what anatomical and evolutionary conditions they become dependent on doing so.

## 5 Cancer cells as donors: immune suppression and niche construction

### 5.1 Tumor-to-T cell transfer and immune corruption

The field initially focused on cancer cells as beneficiaries of organelle donation. More recent studies, however, have made it difficult to sustain such a one-sided view. Tumor cells can also act as mitochondrial donors, and this outward transfer may be no less important biologically. Indeed, if one thinks of the TME as an ecosystem under metabolic pressure, then export of damaged mitochondria by tumor cells is almost as intuitive as import of healthy ones.

The clearest and perhaps most provocative example involves tumor-to-T-cell transfer. Clinical evidence suggests that tumor cells can deliver mitochondria carrying mutant mtDNA into tumor-infiltrating lymphocytes, with some recipient T cells undergoing a dramatic loss of endogenous mitochondrial identity, up to homoplasmic replacement in extreme cases (7). The relevance of this observation extends beyond the novelty of organelle movement. It implies that mitochondrial transfer can directly alter the mitochondrial genotype-function relationship of immune cells within tumors. If verified broadly, this would represent a qualitatively new mechanism of immune suppression: not only inhibition of signaling or nutrient competition, but structural corruption of the organelle system required for long-term T cell competence.

### 5.2 Post-entry survival of tumor-derived mitochondria

The proposed role of USP30 in this process deserves particular attention (7). The idea that tumor-derived mitochondria survive in T cells because they carry a mitophagy-inhibitory factor, while host mitochondria are more readily eliminated in the ROS-rich TME, is one of the most mechanistically interesting models in the field (7). It suggests that transfer is not just a matter of physical movement but of post-entry fitness within a quality-control environment. In our view, this kind of mechanism will ultimately prove more important than the route of transfer itself. Many cells may exchange mitochondria occasionally, but only some transferred organelles will survive the recipient’s selection machinery.

### 5.3 Tumor-to-stroma transfer and underexplored recipients

Tumor-to-stroma transfer provides a second major example. Cancer cells can donate mitochondria to fibroblasts, inducing a hybrid cancer-associated fibroblast state marked by increased OXPHOS, ATP production, and ROS, together with enhanced secretion of extracellular matrix and inflammatory mediators (8). This observation is conceptually significant because it reframes stromal activation. CAF formation has long been explained through cytokines, growth factors, and matrix remodeling signals. Those mechanisms remain valid, but mitochondrial donation suggests that metabolic reprogramming of fibroblasts may be more direct than previously appreciated (60). A tumor cell can, in effect, export part of its organelle state and thereby help generate the stromal phenotype from which it subsequently benefits.

A similar logic may apply to other recipient populations that remain understudied. Dendritic cells, NK cells, endothelial cells, and tissue-resident macrophages are all plausible recipients of tumor-derived mitochondrial material, but the literature is still sparse. This is an area where the field may be biased by technical tractability. T cells are easier to analyze because they are already of great immunological interest. Fibroblasts are easier to culture and image. But it would be a mistake to assume that these are the only biologically relevant recipients.

### 5.4 Burden shedding as a testable hypothesis

Another point deserves emphasis. Export of dysfunctional mitochondria by tumor cells may serve a dual purpose. It can damage recipient cells, but it may also relieve the tumor cell of internal burden. If a cancer cell can offload depolarized, ROS-generating, or otherwise compromised mitochondria, it is not only harming its neighbors; it is improving its own intracellular economy. Such burden shedding could be especially advantageous under chemotherapy, radiotherapy, or hypoxic stress. In this sense, mitochondrial export may be analogous to secretion of toxic metabolites or immunomodulatory vesicles. It externalizes damage while retaining growth potential.

We should be explicit that “burden shedding” is presented here as a working hypothesis rather than an established mechanism. Direct experimental evidence that donor tumor cells gain a measurable fitness advantage specifically through the export of damaged mitochondria—distinct from passive release secondary to stress—is currently limited. What has been demonstrated is that tumor-derived mitochondria delivered to T cells and fibroblasts are often dysfunctional or carry mutant mtDNA (7, 8), and that tumor cells engage in outward transfer under stress conditions (61). What has not been formally tested is whether suppression of outward transfer, while keeping inward transfer intact, impairs donor-cell survival under chemotherapy or hypoxia. Until such loss-of-function experiments are performed, burden shedding should be regarded as an interpretive framework that deserves direct experimental scrutiny rather than a documented physiological program.

### 5.5 Functional mitochondrial donation as pro-tumor niche support

An important alternative interpretation deserves explicit consideration here, and we are indebted to a thoughtful critique for prompting us to develop it. The framing of tumor cells as exporters of dysfunctional mitochondria implicitly assumes that tumor cells possess a surplus of damaged organelles available for discard. This assumption is not always warranted. Actively proliferating tumor cells require sustained mitochondrial biogenesis to support anabolic demand (62, 63), and complete loss of mtDNA abolishes both proliferative and tumorigenic potential until functional mitochondria are reacquired from host stromal cells (64, 65). These findings indicate that malignant cells are under strong selective pressure to preserve intracellular conditions favorable for mitochondrial renewal. In bioenergetic terms, tumor cells are often more, not less, self-sufficient than their normal counterparts (66). An accumulation of dysfunctional organelles within tumor cells would, by contrast, drive excessive ROS production, deplete ATP, and ultimately precipitate cell death (67, 68) — outcomes incompatible with sustained malignant expansion. It follows that the cells most likely to harbor a surplus of damaged mitochondria within the TME are not actively proliferating tumor cells but rather chronically stressed lymphocytes and senescent stromal populations whose biogenesis and mitophagy machinery is itself compromised (38, 69).

This reasoning suggests a complementary model that, we believe, strengthens rather than contradicts the framework developed above. In addition to — or, in many contexts, instead of — exporting damaged organelles, tumor cells may donate functional mitochondria to selected recipients within the TME, particularly to pro-tumor immune populations such as M2-polarized tumor-associated macrophages (TAMs), regulatory T cells (Tregs), and myeloid-derived suppressor cells (MDSCs). The suppressive and tissue-remodeling activities of these populations depend critically on sustained mitochondrial respiration and fatty acid oxidation: M2 macrophage polarization is governed by PGC-1β–driven oxidative metabolism (70, 71); MDSC immunosuppressive function requires fatty acid oxidation (72); and Treg lineage stability and function in lactate-rich, glucose-poor environments depend on oxidative phosphorylation (73, 74). Yet many of these cells operate under chronic inflammatory and oxidative stress that erodes their endogenous mitochondrial fitness. By supplying functional mitochondria to such recipients, tumors could maintain the viability and effector capacity of cells whose continued activity generates pro-tumor inflammation, immune suppression, and stromal remodeling — processes that, in turn, drive tumor expansion (73, 75). Under this view, mitochondrial donation by tumor cells is not principally a damage-shedding mechanism but a form of active niche construction. It also reveals a dual role for functional mitochondria in tumorigenesis: when retained, they sustain the malignant cell itself; when selectively exported, they sustain the supportive cellular ecosystem that surrounds it.

### 5.6 Distinguishing damaged-cargo sabotage from functional-cargo support

The two interpretations — dysfunctional cargo as burden offloading, and functional cargo as ecosystem support — are not mutually exclusive. Which predominates is likely to depend on the donor cell’s metabolic state, the intensity of acute stress, and the identity of the recipient. Therapy-stressed or severely hypoxic tumor subpopulations with overloaded quality-control machinery may shed damaged organelles to T cells or fibroblasts, contributing to immune dysfunction and stromal corruption (7, 42). Conversely, metabolically robust tumor cells in nutrient-replete regions may export functional organelles to pro-tumor leukocytes, securing a permissive niche, in a manner conceptually analogous to the well-documented stromal-to-tumor transfer of healthy mitochondria via tunneling nanotubes and extracellular vesicles (45, 76). Discriminating between these modes in any given system will require explicit measurement of donated mitochondrial quality — membrane potential, mtDNA integrity, and respiratory capacity in the transferred fraction — rather than reliance on transfer detection alone. We regard this as one of the most important methodological priorities for the field, because the same transfer event, depending on cargo quality, may either harm or help antitumor immunity.

**Figure 3:**
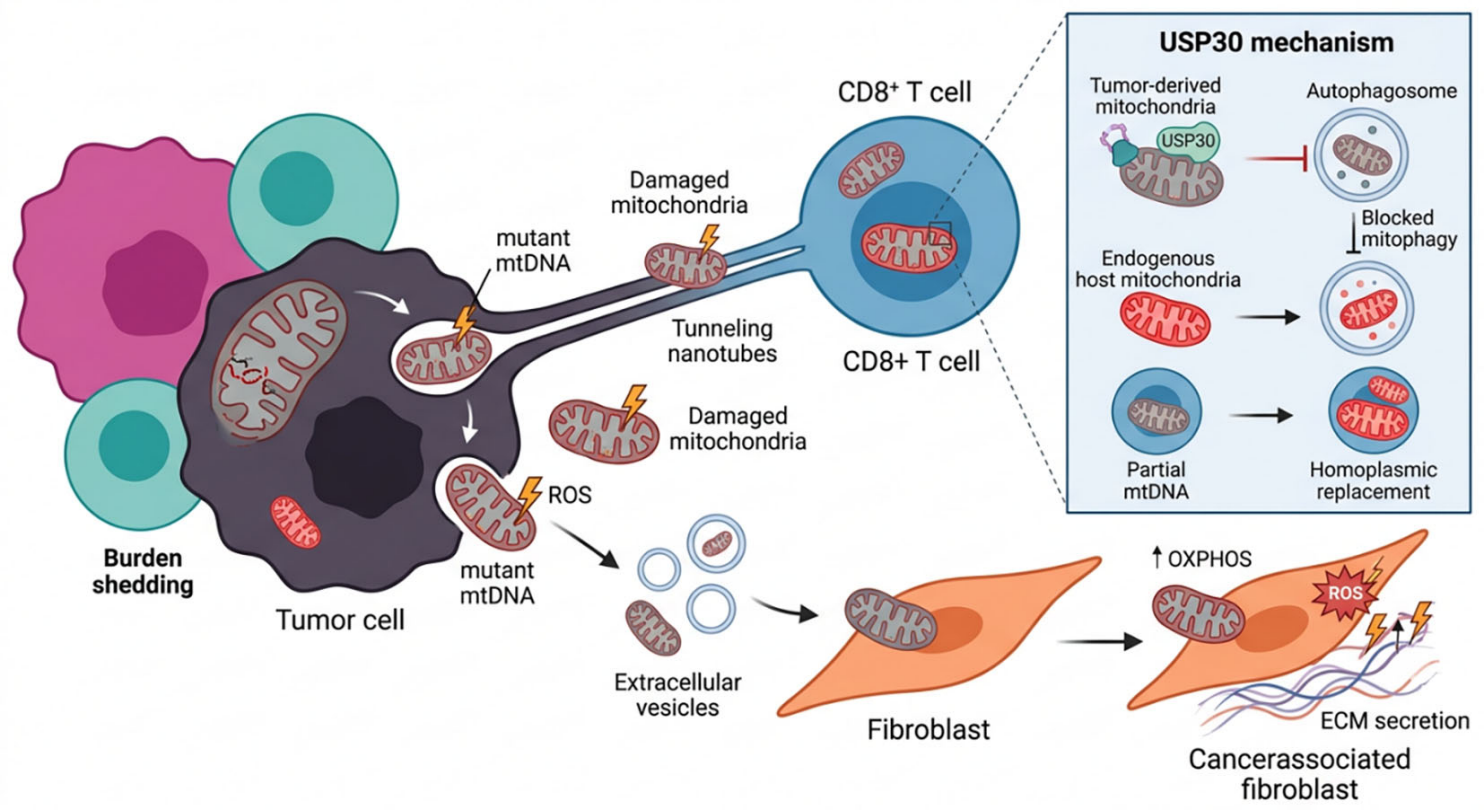
Burden shedding: tumor-derived mitochondria corrupt the microenvironment. Tumor cells export damaged mitochondria carrying mutant mtDNA to CD8^+^ T cells via tunneling nanotubes and to fibroblasts via extracellular vesicles, simultaneously relieving intracellular burden. Inset: the USP30 mechanism — tumor-derived mitochondria evade mitophagy by blocking autophagosome engulfment, while endogenous host mitochondria lacking this protection are selectively degraded, ultimately leading to homoplasmic replacement of the T cell mitochondrial pool. In fibroblasts, uptake of tumor-derived mitochondria drives conversion to cancer-associated fibroblasts (CAFs) with elevated OXPHOS, ROS production, and extracellular matrix secretion.

This perspective may help explain why some studies report directionality opposite to the classic stromal-to-tumor pattern. For example, T-ALL cells have been observed to transfer mitochondria to mesenchymal stromal cells under chemotherapy, reducing ROS in leukemia cells and promoting survival (61). Rather than treating this as an odd exception, it is more coherent to see it as another manifestation of burden redistribution. AML cells, which rely more strongly on OXPHOS, often import mitochondria (20). T-ALL cells, which are more glycolytic, may gain more by exporting damaged mitochondria (61, 77). Directionality, then, reflects metabolic strategy(**Figure 3**). By the same logic, donor tumor cells need not export only damaged organelles; under conditions of preserved metabolic robustness, they may instead export functional mitochondria to recipients whose sustained activity benefits the tumor — a possibility we develop in Section 5.4 as a complementary, recipient-selective mode of donation (75, 78).

There is also a larger conceptual lesson here. The TME is often discussed as though cancer cells are the only active engineers and all other cells are either supportive or defensive responders. Mitochondrial transfer complicates that picture. It reveals a system in which cells are constantly renegotiating who bears the cost of metabolic stress. Tumor cells can receive help, but they can also displace damage. The net result is not simply stronger cancer cells; it is a locally corrupted environment in which surrounding cells become less capable of resisting malignant progression.

## 6 Immune consequences: sabotage, support, and recipient lineage

If there is one area in which mitochondrial transfer may have the most profound conceptual implications, it is tumor immunity. The immunology literature has already established that T cell activation, differentiation, memory formation, and exhaustion are tightly linked to metabolic state (37, 38, 79). What mitochondrial transfer adds is the possibility that this metabolic state is not governed solely by intracellular adaptation, but can be altered directly by neighboring cells.

### 6.1 Mitochondrial loss from cytotoxic T cells

The transfer of mitochondria from T cells to cancer cells has been reported in several experimental systems and appears to depend on contact-dependent structures involving TNTs, actin remodeling, and Miro1-associated trafficking (28, 42). Tumor-derived TNF-α may facilitate this process by promoting TNT formation (28). The functional consequences for T cells are intuitive but still underappreciated: mitochondrial loss deprives them not only of respiratory capacity, but also of spare metabolic reserve, ROS signaling balance, and the mitochondrial fitness needed for long-term function (28, 42). In other words, tumor cells do not merely steal energy; they erode the organelle infrastructure that sustains cytotoxicity.

This matters because exhausted T cells are already metabolically compromised. Chronic antigen exposure, PD-1 signaling, oxidative stress, and nutrient competition all push them toward mitochondrial dysfunction (37, 80). If tumors can siphon mitochondria away from these cells, then organelle theft becomes one more layer in a broader syndrome of metabolic attrition. The fact that T cell exhaustion is hierarchical—from more reversible progenitor-like states to terminally dysfunctional states—raises an important possibility: mitochondrial depletion may help drive the transition from one state to the other. This remains to be proven directly, but it is consistent with what is already known about the role of mitochondrial quality in T cell durability.

### 6.2 Tumor-derived mitochondria entering T cells

The reverse direction of transfer is, if anything, even more provocative. Tumor-derived mitochondria can enter T cells via TNTs or EVs, and those incoming organelles may persist preferentially if they evade mitophagy (7). The notion of homoplasmic replacement in T cells is especially striking because it implies a depth of reprogramming beyond transient suppression. If tumor-derived mutant mitochondria can ultimately dominate the T cell mitochondrial pool, then immune dysfunction is no longer a purely signaling-driven phenomenon; it becomes a form of organelle-level reconstitution. That would help explain why some exhausted states prove so difficult to reverse even with checkpoint blockade.

We think the field should pay much closer attention to the distinction between transient metabolic inhibition and structural mitochondrial replacement. Checkpoint inhibitors can release inhibitory signaling (37), but they may not fully restore immunity if the recipient T cell has already undergone profound organelle damage or replacement. This could be one reason why checkpoint therapy fails in tumors that otherwise appear immunologically infiltrated. It is not enough for T cells to be present. They must retain mitochondria capable of sustaining function.

### 6.3 Immune-supportive mitochondrial transfer

At the same time, the immune consequences of mitochondrial transfer are not uniformly negative. Bone marrow stromal cells can donate mitochondria to CD8+ T cells, improving mitochondrial respiration, proliferation, resistance to apoptosis, tumor infiltration, and persistence through cell division (50). These findings should be taken seriously not only as an experimental curiosity, but as a challenge to the prevailing assumption that mitochondrial transfer in tumors is principally a mechanism of immune suppression. It can be that, but it can also operate in the opposite direction and improve antitumor immunity when donor identity and transfer context differ.

**Figure 4:**
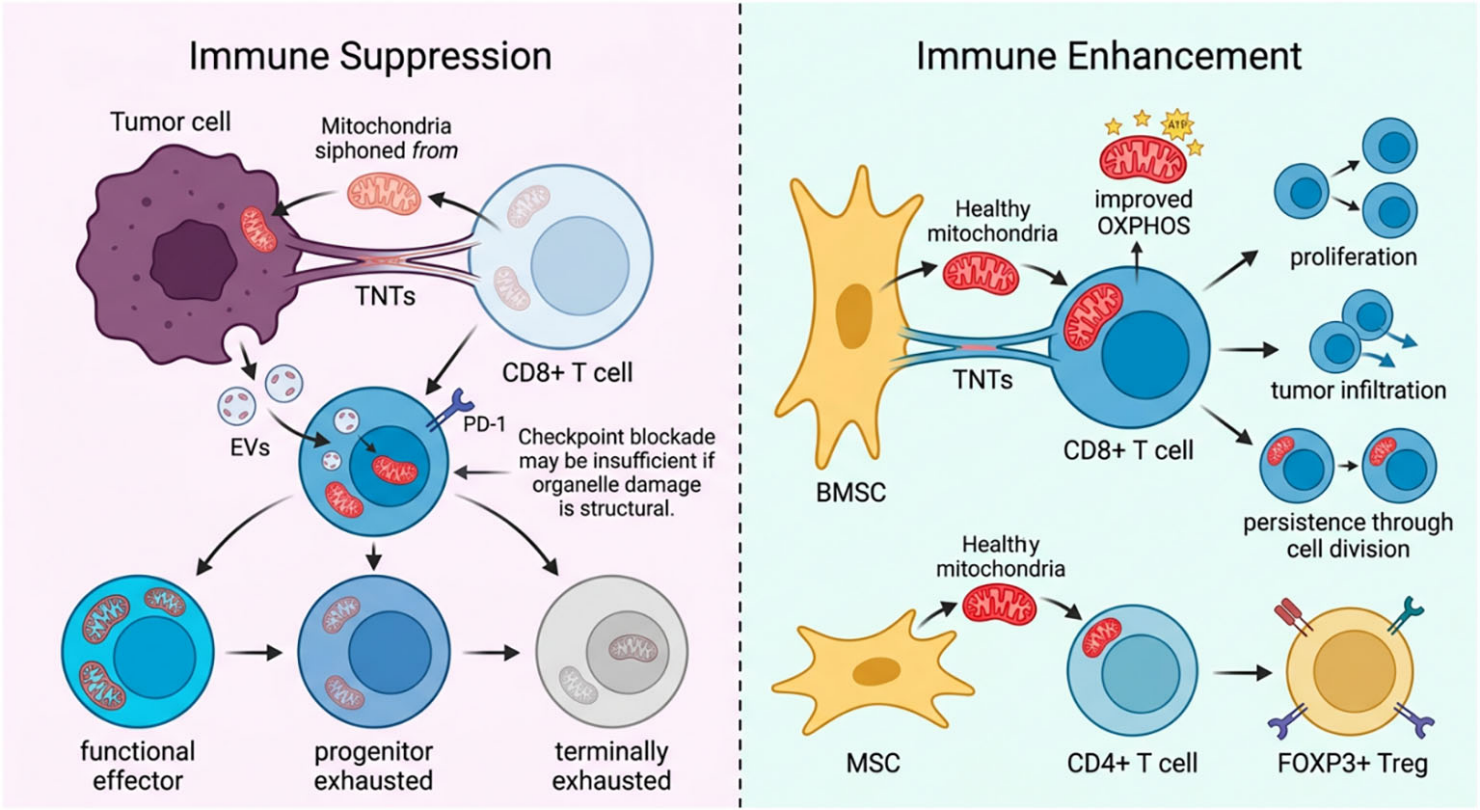
Dual faces of mitochondrial transfer in antitumor immunity. Left: Tumor cells siphon mitochondria from CD8^+^ T cells via TNTs and deliver tumor-derived organelles via EVs, driving progressive exhaustion from functional effector to terminally dysfunctional states with depleted mitochondrial pools. Checkpoint blockade alone may be insufficient when organelle damage is structural. Right: Bone marrow stromal cells (BMSCs) donate healthy mitochondria to CD8^+^ T cells, improving OXPHOS, proliferation, tumor infiltration, and persistence through cell division. Mesenchymal stem cells (MSCs) can also transfer mitochondria to CD4^+^ T cells, promoting FOXP3^+^ regulatory T cell differentiation — highlighting that immune consequences depend critically on donor identity and recipient lineage.

This duality is even more apparent in studies using mesenchymal stem cells. Mitochondrial transfer from MSCs to CD^4+^ T cells can promote regulatory programs involving FOXP3, IL2RA, CTLA4, and TGFβ1, increasing suppressive Treg-like populations (81). In other words, mitochondrial donation can either strengthen effector function or reinforce tolerance depending on the recipient lineage and signaling environment. That is an important warning against simplistic therapeutic proposals. “Boosting T cell mitochondria” is not a single intervention. The biological effect depends on which T cell subset is targeted and what differentiation state it occupies(**Figure 4**).

The broader implication is that mitochondrial transfer may be a hidden variable in tumor immune composition. Two tumors with similar numbers of infiltrating lymphocytes may behave differently if one supports stromal mitochondrial donation to effector T cells while the other promotes tumor-derived mitochondrial sabotage. Current clinical pathology does not measure this. Nor do most single-cell analyses capture organelle origin with enough resolution to reconstruct transfer history. Yet this may turn out to be highly relevant for predicting response to immunotherapy.

Non-T immune cells deserve closer scrutiny. NK cells, dendritic cells, and macrophages all rely on mitochondrial programs for function (10, 82). Some evidence already suggests that mitochondrial transfer influences macrophage polarization and broader innate signaling (83–86). Whether tumors similarly manipulate NK cells or dendritic cells through organelle exchange remains an open but highly plausible question. If so, mitochondrial transfer could be one of the common denominators linking metabolic immune dysfunction across both adaptive and innate compartments.

### 6.4 Recipient-selective mitochondrial support of immunosuppressive cells

A specific possibility deserves dedicated attention. Most existing discussion of tumor-to-immune mitochondrial transfer has emphasized the delivery of damaged organelles to cytotoxic effectors such as CD8^+^ T cells, framing transfer as a sabotage mechanism (42). However, transfer of functional mitochondria to pro-tumor immune populations — including M2-like TAMs, MDSCs, and Tregs — may represent an equally important and currently underappreciated axis of immune evasion. Because the immunosuppressive activity of these populations is metabolically demanding and depends on intact mitochondrial respiration and fatty acid oxidation (71, 74), organelle donation could function as a metabolic license that sustains immunosuppression even when endogenous mitochondrial fitness within stressed leukocytes is declining. This reframes mitochondrial transfer from a purely defensive tactic against effector cells into an active strategy of supportive niche construction within the immune compartment. Direct experimental tests of this hypothesis — for example, comparing the quality (membrane potential, mtDNA integrity) of mitochondria transferred from tumor cells to CD8^+^ T cells versus to TAMs or Tregs in matched tumors — would clarify whether tumors operate a recipient-selective triage system in which damaged organelles are routed to adversaries while functional organelles are routed to allies.

## 7 Tissue ecology of mitochondrial transfer

One of the reasons the field has advanced quickly is that it no longer treats the TME as metabolically generic. Different tissues provide different donor populations, and these donor identities shape the meaning of mitochondrial transfer. This point deserves emphasis because it helps explain why findings from leukemia, glioblastoma, prostate cancer, bone metastasis, and lung cancer should not be expected to align perfectly.

### 7.1 Bone marrow niche

Bone marrow stromal cells are among the most intensively studied donors, particularly in hematologic malignancies. In AML, they transfer mitochondria to leukemic blasts, enhancing ATP generation and OXPHOS and contributing to therapy resistance (20, 45). This interaction is not incidental; it reflects the intimate physical and functional architecture of the marrow niche. The marrow is a setting in which leukemic cells and stromal cells maintain close contact, making TNT-mediated exchange highly plausible. Importantly, inhibition of NOX2 reduces this transfer and promotes AML apoptosis, suggesting that oxidative stress signaling in the leukemia cells actively solicits mitochondrial support (45). This is a powerful example of how malignant cells can co-opt a normal stromal reserve.

The same marrow compartment can, however, support immunity under different conditions. BMSCs can enhance CD8+ T cell function through mitochondrial donation (50). This observation should prevent the field from adopting a binary view of “good” and “bad” stromal cells. The same donor population may support either malignant survival or immune competence depending on the recipient and the cytokine context. This complicates any therapeutic strategy aimed at globally suppressing stromal transfer.

### 7.2 Fibroblast and endothelial donors in solid tumors

In solid tumors, fibroblasts and endothelial cells are often the more relevant donors. Endothelial-to-tumor mitochondrial transfer has been linked to doxorubicin resistance in breast cancer (27), while CAF-to-tumor transfer can reinforce oxidative metabolism and aggressive behavior in prostate and other cancers (46). These interactions reflect the fact that stromal cells are not simply nutrient providers. They can function as mobile metabolic reservoirs whose organelles are mobilized under stress. Whether all stromal donors are equally permissive remains doubtful; donor age, activation state, and mitochondrial quality are likely to matter greatly (87–89).

### 7.3 Tissue-specific donors and organotropism

Tissue-specific donors introduce an additional layer of complexity. Neurons can donate mitochondria to tumor cells and thereby enhance stemness, metabolic fitness, and metastatic capacity (19). Astrocytes can transfer mitochondria to glioblastoma cells (55). These observations are particularly important because they suggest that mitochondrial transfer may contribute to organotropism. Certain tissues may be permissive metastatic sites not merely because of soluble factors or immune tolerance, but because they contain cell types capable of providing compatible mitochondrial support. That possibility deserves far more attention than it has received.

By contrast, osteocyte-to-tumor transfer in bone metastasis appears to activate cGAS/STING signaling and restrain tumor progression (22). This study is important precisely because it resists the dominant narrative. It shows that mitochondrial transfer does not inevitably support cancer and that tissue-specific donor-recipient pairings may produce qualitatively different immune consequences. In our opinion, this is one of the most conceptually useful findings in the recent literature because it forces the field to think beyond transfer quantity and focus on transfer meaning.

### 7.4 Macrophages as mobile donors and recipients

Macrophages occupy a special position because they are both highly plastic and highly mobile. They can receive damaged mitochondria through transmitophagy-like processes (84, 90), but they can also donate mitochondria to surrounding cells. In tumors, M2-like macrophage-derived mitochondria may serve as ROS-generating signals that enhance cancer cell proliferation (91, 92). However, the macrophage compartment is heterogeneous, and the functional state of donor mitochondria is likely to differ across inflammatory contexts. The field would benefit from distinguishing whether it is the macrophage lineage or the macrophage polarization state that determines donor behavior.

The broader message is that mitochondrial transfer cannot be understood independently of tissue ecology. The donor cell repertoire in marrow is not the same as in brain, bone, or peritoneum. Nor are the physical opportunities for transfer equivalent. This may explain why some cancers appear especially dependent on mitochondrial exchange while others do not. It may also explain why therapeutic disruption of transfer could prove highly context-specific.

## 8 Mechanistic pathways: what we know, and what we still do not

Considerable progress has been made in identifying routes of mitochondrial transfer, yet one gets the sense that the field sometimes confuses structural description with mechanistic explanation. We know a fair amount about how mitochondria can move from one cell to another. We know far less about why a given transfer event is stabilized, retained, and made biologically consequential.

### 8.1 Tunneling nanotubes

TNTs remain the best-characterized route (93, 94). They provide direct cytoplasmic continuity and are especially suited to transfer of intact organelles. Their formation depends on actin remodeling and is promoted by stresses common in tumors, including hypoxia, ROS, inflammatory cytokines, and treatment-induced damage (48, 49, 59). Molecular regulators such as MIRO1/RHOT1, MIRO2, DRP1, MTFR2, RAC1, CDC42, HMGB1, TNFAIP2, and related cytoskeletal pathways have all been implicated (95–98). They show that mitochondrial transfer is not a mystical process but one integrated into known networks of organelle dynamics and actin regulation.

However, TNT biology alone does not solve the more important question of transfer selectivity. Why does one cell extend a productive nanotube to a particular partner and not another? Why do some TNT-associated transfer events restore respiration, while others deliver dysfunctional organelles that sabotage recipient cells? The field often implies that stress simply increases TNT abundance and therefore transfer frequency. That is probably true, but it is insufficient. A useful mechanistic model must explain polarity and cargo choice. At present, these remain underdeveloped areas.

### 8.2 Extracellular vesicles

Extracellular vesicles represent a second major route (13–15). They are attractive conceptually because they allow transfer without prolonged cell-cell contact and may operate over greater distances within dense tissues. Yet EV-mediated mitochondrial transfer is also easy to overinterpret. Not all EV-associated mitochondrial material is functionally equivalent. Some vesicles carry mtDNA fragments, some contain mitochondrial proteins, and some may contain more intact organellar structures. The biological consequences of these cargos are unlikely to be identical. Unfortunately, many studies still classify them together. A more careful nomenclature would improve clarity.

### 8.3 Cell fusion and uptake of extracellular mitochondria

Cell fusion, gap junction-related processes, and uptake of extracellular mitochondria through endocytic or phagocytic routes add further complexity (52, 99–101). These mechanisms are certainly real, but their quantitative contribution in most tumors remains poorly defined. The field occasionally lists them all as if they were equally important in every setting. In our view, the more mature approach is to ask which route is favored by which anatomical context. TNTs may dominate in tightly apposed niches such as bone marrow. EV-mediated routes may be more important in complex solid tumors. Cell fusion may be rare but disproportionately consequential when it occurs. Recent work by Qiao et al. in adenoid cystic carcinoma reinforces the view that mitochondrial transfer and cell fusion are mechanistically linked rather than independent routes (102). In their system, TNT-mediated transfer dominates early while permanent fusion emerges later, and the two share upstream triggers—recipient mitochondrial dysfunction, L-lactate–driven TNT recruitment, and TMEM16F-mediated phosphatidylserine externalization. Notably, fusion produced hybrid cells with enhanced EMT and invasiveness beyond what mitochondrial transfer alone conferred, suggesting that the two modalities lie on a temporal continuum in which fusion represents a more consequential endpoint when intercellular contact persists.

### 8.4 Donor-recipient specificity

A more fundamental question is why specific donor-recipient pairs form in the first place. Several determinants are beginning to emerge. First, electrochemical asymmetry between cells appears to bias direction: recipient cells frequently display lower mitochondrial membrane potential (ΔΨm), reduced ATP/ADP ratios, or accumulated ROS, whereas donors tend to retain polarized, fusion-competent mitochondrial networks (45, 50). NOX2-derived superoxide in AML blasts, for instance, functions as a recruitment signal that actively solicits stromal donation, suggesting that recipient-derived oxidative cues, rather than donor altruism, may initiate pairing (45). Second, adhesion and contact molecules provide physical specificity. ICAM-1/VCAM-1, integrin-mediated coupling, CD38, and connexin-43 (CX43) gap junctions have all been implicated in stabilizing donor-recipient interfaces and licensing organelle transit (49, 100, 103). Third, lineage-restricted scaffold proteins—GAP43 in astrocytes, TNFAIP2/M-Sec in stromal cells, and MIRO2 in tumor cells—appear to confer cell-type-specific competence to extend or receive TNTs (8, 54, 94). Together, these observations argue that donor-recipient specificity is not random proximity but a regulated negotiation: a metabolically stressed recipient broadcasts a demand signal, while only cells expressing the appropriate adhesion and trafficking machinery can answer it. This framework also predicts that disruption of either the demand signal (e.g., NOX2 inhibition) or the structural interface (e.g., CX43 blockade) should selectively impair pathological transfer while sparing constitutive exchange.

### 8.5 Activation and apoptosis as polarity cues

Beyond bioenergetic asymmetry and adhesion-mediated specificity, two additional classes of stimuli deserve explicit attention because they appear to act not only as triggers but as polarity-determining cues: cell activation and apoptosis. Activation-coupled transfer has been documented across multiple donor lineages. Activated platelets release functional mitochondria, either as free organelles or encapsulated within microparticles, and these can be taken up by neutrophils, endothelial cells, and tumor cells, often with pro-inflammatory or pro-survival consequences (104, 105). Inflammatory activation of mesenchymal stem cells by LPS, IFN-γ, or TNF-α enhances their capacity to extend TNTs and donate mitochondria to stressed recipients, including injured epithelial and immune cells (106, 107). T cell receptor engagement itself induces transient mitochondrial remodeling and increases the likelihood of contact-dependent organelle exchange with antigen-presenting cells and tumor targets (50, 108). These observations indicate that activation does not merely raise the probability of transfer; it specifies which cells become competent donors and toward which partners they project organelles.

Apoptosis introduces a complementary and equally directional layer of control. Early apoptotic signaling externalizes phosphatidylserine through TMEM16F-mediated scrambling, a step that has been shown to both license TNT formation and mark cells for engulfment by neighboring phagocytes (106, 109). In adenoid cystic carcinoma, TMEM16F-dependent phosphatidylserine externalization couples mitochondrial transfer with subsequent cell fusion, illustrating that the same apoptotic-adjacent signal can determine both the route and the persistence of organelle exchange (106). Caspase-dependent processes can additionally trigger mitochondrial release into the extracellular space or packaging into apoptotic bodies, which are then captured by surrounding cells — effectively converting cell death into a vehicle for organelle redistribution (110, 111). Crucially, apoptotic and pre-apoptotic cells tend to act as donors rather than recipients, biasing transfer outward and toward viable neighbors with intact phagocytic or endocytic machinery. This is biologically intuitive: cells facing terminal stress are unlikely to benefit from incoming organelles, whereas their release of mitochondrial cargo may support adjacent cells or, in pathological contexts, propagate damage-associated signals.

Taken together, activation and apoptosis should be regarded as directional inputs to the organelle economy rather than nonspecific stress triggers. Activation typically licenses donor competence; apoptosis typically enforces outward polarity. Both integrate with the membrane-potential and adhesion-based asymmetries discussed above, and both help explain why transfer direction in the TME is so context-dependent. We suggest that future mechanistic studies explicitly stratify donor and recipient populations by activation state and apoptotic status, because these variables may resolve apparent inconsistencies in reported transfer polarity more effectively than cell-type identity alone.

### 8.6 Cargo selection before export

An equally underdeveloped question is what determines which mitochondria are loaded for export. Cargo selection appears to be neither random nor uniform. DRP1-mediated fission generates smaller mitochondrial fragments that are more readily packaged into TNT lumens or budding vesicles, and MTFR2-dependent fission has been shown to selectively mobilize fatty acid–laden mitochondria from hepatic stellate cells to tumor cells (96, 98). MIRO1 and MIRO2 act as molecular adaptors coupling specific mitochondrial subpopulations to kinesin-driven transport, with MIRO2 in particular implicated in directing damaged mitochondria from tumor cells toward fibroblast recipients (8, 42). Pre-export quality control also plays a role: PINK1/Parkin marking can paradoxically channel depolarized mitochondria into the secretory pathway rather than into lysosomal degradation, effectively converting mitophagy substrates into exportable cargo under conditions where autophagic flux is saturated (51). Conversely, healthy mitochondria selected for stromal-to-T cell donation appear to be those with high membrane potential and intact cristae, consistent with their capacity to be retained and replicate in the recipient (50). The implication is that donor cells operate a triage system in which mitochondrial quality, fission status, and adaptor availability collectively determine which organelles leave the cell and through which route. The biological consequence of any transfer event is therefore pre-specified, at least in part, before the cargo ever reaches the recipient.

### 8.7 Post-transfer fate checkpoints

The real mechanistic frontier, however, lies downstream of transport. Mitochondria entering a cell face a gauntlet of quality-control processes that collectively determine whether the incoming organelle becomes a biological asset, an inert passenger, or a substrate for degradation. The first checkpoint is PINK1/Parkin-mediated mitophagy. Incoming mitochondria with reduced ΔΨm stabilize PINK1 on the outer membrane, recruit Parkin, and are tagged with K48- and K63-linked ubiquitin chains that direct them to autophagosomes (51, 112). Whether a transferred mitochondrion clears this checkpoint depends not only on its intrinsic membrane potential but on the recipient’s autophagic capacity at the moment of arrival—a parameter that varies dramatically between activated T cells, quiescent stromal cells, and stressed tumor cells. The second checkpoint is deubiquitination-mediated rescue. USP30, an outer-membrane deubiquitinase that opposes Parkin-driven ubiquitination, can tip the balance from degradation to retention (7, 113). The observation that tumor-derived mitochondria entering T cells carry elevated USP30 activity provides a mechanistically coherent explanation for homoplasmic replacement: incoming organelles actively neutralize the recipient’s mitophagy machinery while endogenous host mitochondria, lacking equivalent protection in the ROS-rich tumor microenvironment, are preferentially cleared (7). The third checkpoint is integration via fusion. Retained mitochondria must fuse with the host network through MFN1/MFN2 and OPA1-mediated events to share matrix contents, exchange mtDNA nucleoids, and become subject to coordinated biogenesis (50, 51, 114). Fusion failure leaves transferred organelles as isolated, non-replicating units with limited durable impact, even if they escape immediate degradation. The fourth and most rigorous checkpoint is replication and heritability. Only mitochondria that successfully fuse, undergo TFAM-dependent mtDNA replication, and segregate through cell division can be considered functionally established (115). Direct evidence of this final stage—daughter-cell inheritance of donor-derived mitochondria in CD8^+^ T cells (50) and homoplasmic mtDNA replacement in tumor-infiltrating lymphocytes (7)—remains rare in the literature precisely because it is the most demanding criterion to satisfy. We suspect that this layered post-transfer selection, rather than the route of transport, will prove central to the field’s future. It also predicts that pharmacological modulation of USP30, Parkin, or MFN1/2 may offer a more selective therapeutic axis than blocking transfer routes themselves, because it targets the step at which biological consequence is actually decided.

### 8.8 Methodological limitations in proving durable integration

**Figure 5:**
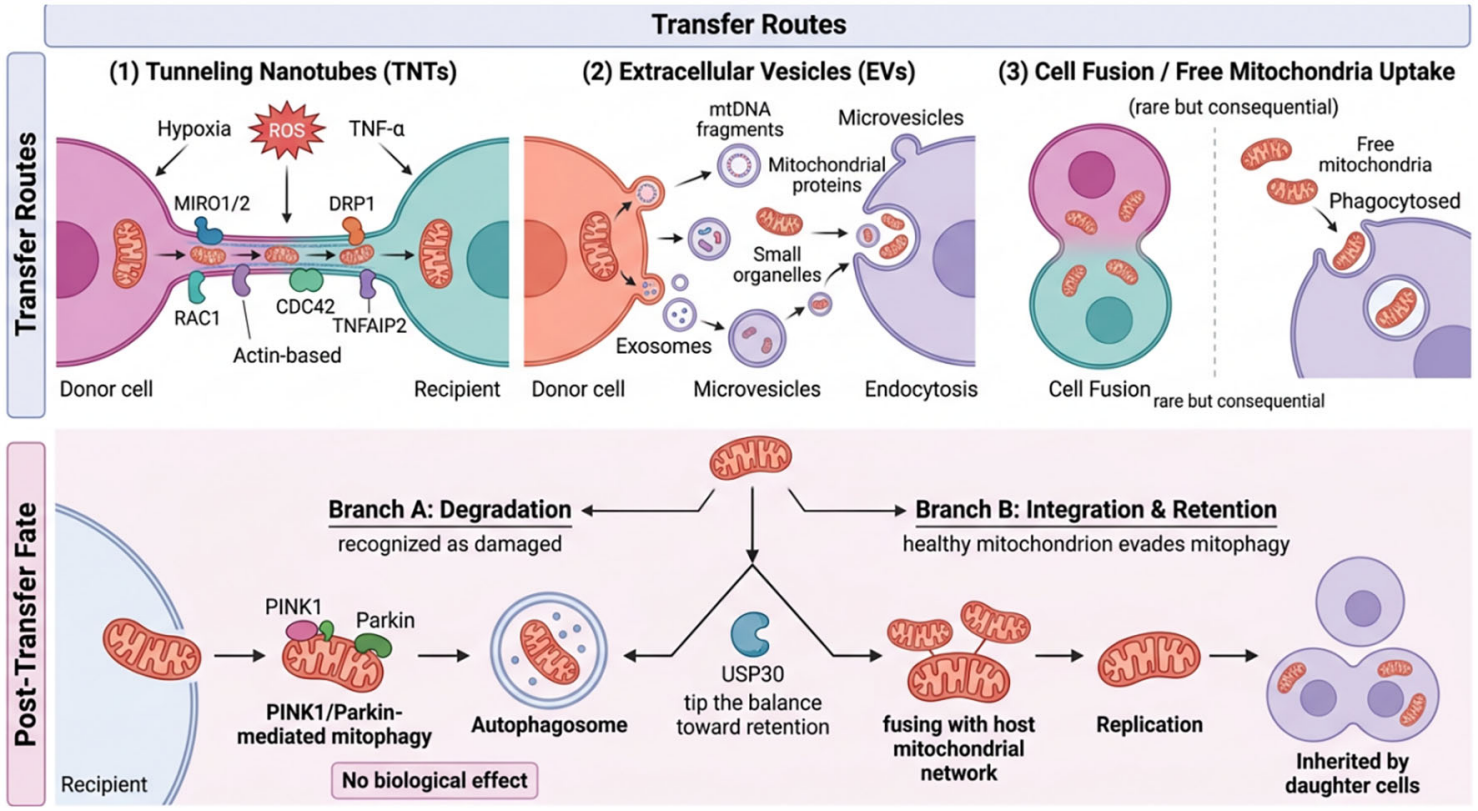
Transfer routes and post-transfer fate: the critical quality control checkpoint. Upper panel: Three principal transfer routes — (1) tunneling nanotubes (TNTs), formed via actin-based remodeling MIRO1/2,DRP1,RAC1,CDC42,TNFAIP2 under hypoxia, ROS, and TNF-α stimulation; (2) extracellular vesicles carrying mtDNA fragments, mitochondrial proteins, or small organelles; (3) cell fusion and phagocytic uptake of free mitochondria. Lower panel: Post-transfer fate decision — incoming mitochondria either undergo PINK1/Parkin-mediated mitophagy (degradation, no biological effect) or evade clearance, aided by USP30, fusing into the host network, replicating, and being inherited by daughter cells. This retention checkpoint ultimately determines whether transfer becomes biologically consequential.

The problem is methodological. It is easier to image transfer than to prove stable functional integration. Many published studies do the former and infer the latter (4). That is understandable, but it means the literature may overestimate how often transferred mitochondria become durable biological assets. The studies showing daughter-cell inheritance in T cells (50) or homoplasmic replacement (7) are therefore especially important: they move the field from uptake to persistence. More such work is needed. In short, the current mechanistic map is informative but incomplete. We know many of the roads; we do not yet know the full rules of traffic (**Figure 5**).

## 9 Evidence standards, therapeutic implications, and future perspectives

A recurring difficulty in this field is that the strongest claims are often made on the basis of the least clinically grounded evidence. Mitochondrial transfer is visually compelling—fluorescently labeled organelles moving between cells are hard to ignore—but compelling imagery is not the same as clinically meaningful biology. Having surveyed the mechanistic and biological landscape of mitochondrial transfer, we now turn to the standards by which evidence in this field should be judged, the therapeutic strategies that follow from it, and the conceptual outlook for the next phase of research.

### 9.1 Current evidence base and methodological limitations

Most foundational studies rely on coculture systems, genetically marked donor-recipient pairs, or high-transfer experimental contexts (5, 6). These systems have been extraordinarily informative but also make transfer easier to detect than it may be in patient tissues. In clinical samples, one must distinguish genuine intercellular transfer from cell fusion, phagocytic uptake of debris, imaging artifacts, or contamination by extracellular mitochondrial fragments (3, 4). The field still lacks universally accepted standards for what constitutes proof of functionally significant transfer in human tumors. Compounding this, the number of mitochondria transferred is often low relative to the recipient’s total mitochondrial pool, so functional impact must be demonstrated through respiratory measurements, ROS analysis, fate mapping, and ideally longitudinal assessment of persistence (76). The field would benefit from a more disciplined distinction between “transfer observed” and “transfer biologically decisive”.

Computational approaches such as MERCI represent an important step forward by reconstructing donor-recipient relationships from transcriptomic and mitochondrial patterns, and the observation that transfer-associated phenotypes correlate with hypoxia, cell-cycle activity, and poor outcome across multiple cancers is intriguing (28). However, computational transfer scores remain proxies that do not necessarily distinguish intact mitochondrial exchange from mtDNA transfer or broader stress responses; they are hypothesis-generating, not definitive. More soberingly, despite the rapid expansion of preclinical evidence, no clinical trial has yet directly demonstrated organelle exchange between cancer cells and surrounding compartments in human tumors, nor shown that pharmacological modulation of this process alters tumor behavior in patients (116). The oncology literature offers inferences—correlations between transfer-associated signatures and outcome, retrospective marker detection, computational reconstructions—but these are indirect. The field has therefore reached an asymmetric maturity in which mechanistic biology has advanced considerably faster than the clinical evidence that would justify therapeutic intervention specifically targeting transfer in oncology.

A related issue is timescale. Some transfer events may be acute stress responses with transient consequences, while others induce durable phenotypic shifts through retention across cell division or genotype replacement (7, 50). These are not biologically equivalent, yet many studies present them in the same conceptual frame. Similarly, the relationship between mitochondrial transfer and mtDNA-related phenomena such as numtogenesis remains an area where speculation outpaces direct evidence (17, 18, 117). No study has yet causally demonstrated that intercellular transfer events generate NUMTs in tumors; the two phenomena may share upstream stressors without being mechanistically coupled. Such hypotheses should be presented with explicit acknowledgement of their conjectural status until dedicated longitudinal studies are performed.

### 9.2 Therapeutic strategies: from broad inhibition to context-selective modulation

The therapeutic implications of mitochondrial transfer are obvious enough to be attractive and subtle enough to be dangerous. If tumor cells rely on organelle acquisition to restore metabolism and resist therapy, blocking that process should be beneficial; but if immune or reparative cells also depend on mitochondrial exchange, indiscriminate inhibition could be counterproductive. The challenge is not simply intervention, but selectivity.

One strategy is to reduce the value of transferred mitochondria by suppressing mitochondrial metabolism in recipient tumor cells. Metformin has often been invoked in this regard (118, 119), but the negative phase 2 trial combining metformin with PD-1 blockade is instructive (120): broad metabolic interventions may fail without biomarker-defined patient selection. More direct approaches target transfer routes themselves. Drugs affecting actin or microtubule dynamics can suppress TNT formation (121); farnesyltransferase/geranylgeranyltransferase inhibition reduces Miro-dependent transfer (42); adhesion molecules such as ICAM-1 and VCAM-1 influence donor-recipient contact (103, 122, 123); and NOX2 inhibition reduces stromal-to-AML transfer and improves survival in mouse models (45). EV-mediated transfer presents another targetable route, with potential synergy with anti-PD-1 therapy in immunotherapy-resistant settings. The principal limitation across these approaches is specificity—most available inhibitors affect broader cytoskeletal, signaling, or vesicular processes that could impair immune cell migration, hematopoiesis, or tissue repair.

The more intriguing therapeutic possibility lies in selective mitochondrial support of immune effectors. BMSC-to-CD8^+^ T cell mitochondrial donation enhances T cell persistence, expansion, tumor infiltration, and responsiveness to checkpoint blockade (124), opening a fresh approach to adoptive cell therapy that engineers not only receptor specificity but metabolic durability. This aligns with broader efforts to improve T cell mitochondrial fitness using bezafibrate (125, 126), spermidine (127), and related agents. Artificial mitochondrial transfer and transplantation technologies extend this logic beyond natural donor-recipient systems (128–132), with promising results in ischemic injury, inflammatory disease, aged T cell dysfunction, and cardiovascular repair (51, 133, 134). The growing clinical trial portfolio in non-oncology indications (116) makes it plausible that organelle-based therapy will reach oncology, though direct clinical validation in tumors remains a critical and currently unmet prerequisite.

Yet the risks are substantial. Donor mitochondria are not immunologically inert—mtDNA, formyl peptides, and cardiolipin can activate innate immune pathways (135, 136), and dysfunctional donor mitochondria may worsen recipient injury rather than relieve it (52, 137). Donor quality, including donor cell age and metabolic state (87–89), is therefore central to safety and efficacy. For these reasons, the most promising translational path is not global enhancement or suppression of mitochondrial transfer, but context-selective modulation: blocking donor-recipient coupling where tumor cells depend on stromal mitochondrial support, while controlled augmentation may increase T cell durability in adoptive immunotherapy. Success will depend on a much more precise understanding of transfer polarity, cargo quality, and recipient selection than the field currently possesses.

### 9.3 Conclusions and outlook

Mitochondrial transfer has matured from an intriguing observation into a serious conceptual issue for cancer biology, forcing reconsideration of three assumptions: that mitochondrial competence is cell-autonomous, that tumor metabolic state is determined solely by intrinsic factors, and that immune dysfunction reflects only inhibitory signaling and nutrient competition. What is taking place in the TME is often closer to negotiation, appropriation, and burden shifting than to simple “donation” or “rescue.” Tumor cells may appropriate healthy mitochondria from surrounding cells while displacing defective ones into immune or stromal compartments; stromal cells may support either tumor survival or immune fitness depending on recipient identity; T cells may be either victims of mitochondrial theft or beneficiaries of mitochondrial reinforcement. The process is not morally polarized in biological terms—it is strategically contingent.

We acknowledge that several conceptual devices used throughout this review—organelle economy, burden shedding, ecological exchange, post-transfer fitness checkpoint—are integrative interpretations rather than directly validated mechanisms. Their value is heuristic: they generate testable predictions (for example, that selective inhibition of outward versus inward transfer should produce dissociable phenotypes) that we hope will guide the next generation of experimental work. The field must therefore resist two opposite temptations: romanticizing mitochondrial transfer as a universal repair mechanism, and treating all transfer as harmful and worthy of blanket inhibition. Both views ignore the recipient-, cargo-, and context-dependence that defines this biology.

Two questions remain central. First, what determines transfer direction? The answer likely integrates mitochondrial membrane potential, redox imbalance, cytoskeletal tension, inflammatory cues, and quality-control asymmetry after uptake (138–140). Second, what defines biological relevance? Many studies show transfer; far fewer show stable integration, daughter-cell inheritance, or genotype replacement (7, 50). These distinctions will matter greatly if biomarkers and therapies are to be built on this biology. Nonetheless, mitochondrial transfer is unlikely to fade as a passing trend, because it addresses phenomena that traditional models have struggled to explain—rapid metabolic rescue, durable immune dysfunction, stromal conversion, and persistence of treatment-resistant phenotypes—and provides a plausible mechanism by which these changes can occur without waiting for *de novo* genetic evolution. The next phase of the field should focus less on proving that transfer occurs and more on defining when it matters, when it dominates, and when it can be manipulated to clinical advantage.

## References

[1] Spinelli JB Haigis MC . The multifaceted contributions of mitochondria to cellular metabolism. Nat Cell Biol. (2018) 20:745–54. doi: 10.1038/s41556-018-0124-1. PMID: 29950572 PMC6541229

[2] Vasan K Werner M Chandel NS . Mitochondrial metabolism as a target for cancer therapy. Cell Metab. (2020) 32:341–52. doi: 10.1016/j.cmet.2020.06.019. PMID: 32668195 PMC7483781

[3] Brestoff JR Singh KK Aquilano K Becker LB Berridge MV Boilard E . Recommendations for mitochondria transfer and transplantation nomenclature and characterization. Nat Metab. (2025) 7:53–67. doi: 10.1038/s42255-024-01200-x. PMID: 39820558

[4] Tiash S Brestoff JR Crewe C . A guide to studying mitochondria transfer. Nat Cell Biol. (2023) 25:1551–3. doi: 10.1038/s41556-023-01246-1. PMID: 37853133 PMC11610514

[5] Tan AS Baty JW Dong L-F Bezawork-Geleta A Endaya B Goodwin J . Mitochondrial genome acquisition restores respiratory function and tumorigenic potential of cancer cells without mitochondrial DNA. Cell Metab. (2015) 21:81–94. doi: 10.1016/j.cmet.2014.12.003. PMID: 25565207

[6] Dong L-F Kovarova J Bajzikova M Bezawork-Geleta A Svec D Endaya B . Horizontal transfer of whole mitochondria restores tumorigenic potential in mitochondrial DNA-deficient cancer cells. Elife. (2017) 6:e22187. doi: 10.7554/eLife.22187. PMID: 28195532 PMC5367896

[7] Ikeda H Kawase K Nishi T Watanabe T Takenaga K Inozume T . Immune evasion through mitochondrial transfer in the tumour microenvironment. Nature. (2025) 638:225–36. doi: 10.1038/s41586-024-08439-0. PMID: 39843734 PMC11798832

[8] Cangkrama M Liu H Wu X Yates J Whipman J Gäbelein CG . MIRO2-mediated mitochondrial transfer from cancer cells induces cancer-associated fibroblast differentiation. Nat Cancer. (2025) 6:1714–33. doi: 10.1038/s43018-025-01038-6. PMID: 40877413 PMC12559006

[9] Stine ZE Schug ZT Salvino JM Dang CV . Targeting cancer metabolism in the era of precision oncology. Nat Rev Drug Discov. (2022) 21:141–62. doi: 10.1038/s41573-021-00339-6. PMID: 34862480 PMC8641543

[10] O’Sullivan D Sanin DE Pearce EJ Pearce EL . Metabolic interventions in the immune response to cancer. Nat Rev Immunol. (2019) 19:324–35. doi: 10.1038/s41577-019-0140-9. PMID: 30820043

[11] Reina-Campos M Scharping NE Goldrath AW . CD8(+) T cell metabolism in infection and cancer. Nat Rev Immunol. (2021) 21:718–38. doi: 10.1038/s41577-021-00537-8. PMID: 33981085 PMC8806153

[12] Sena LA Li S Jairaman A Prakriya M Ezponda T Hildeman DA . Mitochondria are required for antigen-specific T cell activation through reactive oxygen species signaling. Immunity. (2013) 38:225–36. doi: 10.1016/j.immuni.2012.10.020. PMID: 23415911 PMC3582741

[13] Iorio R Petricca S Di Emidio G Falone S Tatone C . Mitochondrial extracellular vesicles (mitoEVs): Emerging mediators of cell-to-cell communication in health, aging and age-related diseases. Ageing Res Rev. (2024) 101:102522. doi: 10.1016/j.arr.2024.102522. PMID: 39369800

[14] Kong J Sun R Du C Tang Y Xie C Li Q . Mitochondrial extracellular vesicles: A novel approach to mitochondrial quality control. Biomolecules. (2025) 15:1145. doi: 10.3390/biom15081145. PMID: 40867590 PMC12384377

[15] Peng X Gao Y Liu J Shi X Li W Ma Y . Mitochondria-derived vesicles: A promising and potential target for tumour therapy. Clin Transl Med. (2025) 15:e70320. doi: 10.1002/ctm2.70320. PMID: 40356246 PMC12069804

[16] Singh KK Choudhury AR Tiwari HK . Numtogenesis as a mechanism for development of cancer. Semin Cancer Biol. (2017) 47:101–9. doi: 10.1016/j.semcancer.2017.05.003. PMID: 28511886 PMC5683947

[17] Milano AF . 20-year comparative survival and mortality of cancer of the stomach by age, sex, race, stage, grade, cohort entry time-period, disease duration & selected ICD-O-3 oncologic phenotypes: A systematic review of 157,258 cases for diagnosis years 1973-2014. J Insur Med. (2019) 48:5–23. doi: 10.17849/insm-48-1-1-19.1. PMID: 31609640

[18] Koo D-H Singh B Jiang J Friebe B Gill BS Chastain PD . Single molecule mtDNA fiber FISH for analyzing numtogenesis. Anal Biochem. (2018) 552:45–9. doi: 10.1016/j.ab.2017.03.015. PMID: 28322800 PMC5814351

[19] Hoover G Gilbert S Curley O Obellianne C Lin MT Hixson W . Nerve-to-cancer transfer of mitochondria during cancer metastasis. Nature. (2025) 644:252–62. doi: 10.1038/s41586-025-09176-8. PMID: 40562940 PMC12328229

[20] Moschoi R Imbert V Nebout M Chiche J Mary D Prebet T . Protective mitochondrial transfer from bone marrow stromal cells to acute myeloid leukemic cells during chemotherapy. Blood. (2016) 128:253–64. doi: 10.1182/blood-2015-07-655860. PMID: 27257182

[21] Kidwell CU Casalini JR Pradeep S Scherer SD Greiner D Bayik D . Transferred mitochondria accumulate reactive oxygen species, proliferation. Elife. (2023) 12:e85494. doi: 10.7554/eLife.85494. PMID: 36876914 PMC10042539

[22] Zhou H Zhang W Li H Xu F Yinwang E Xue Y . Osteocyte mitochondria inhibit tumor development via STING-dependent antitumor immunity. Sci Adv. (2024) 10:eadi4298. doi: 10.1126/sciadv.adi4298. PMID: 38232158 PMC10793952

[23] King MP Attardi G . Injection of mitochondria into human cells leads to a rapid replacement of the endogenous mitochondrial DNA. Cell. (1988) 52:811–9. doi: 10.1016/0092-8674(88)90423-0. PMID: 3349520

[24] Wu T-H Sagullo E Case D Zheng X Li Y Hong JS . Mitochondrial transfer by photothermal nanoblade restores metabolite profile in mammalian cells. Cell Metab. (2016) 23:921–9. doi: 10.1016/j.cmet.2016.04.007. PMID: 27166949 PMC5062745

[25] Lee AR Woo JS Lee S-Y Na HS Cho K-H Lee YS . Mitochondrial transplantation ameliorates the development and progression of osteoarthritis. Immune Netw. (2022) 22:e14. doi: 10.4110/in.2022.22.e14. PMID: 35573148 PMC9066007

[26] Lee JM Hwang JW Kim MJ Jung SY Kim K-S Ahn EH . Mitochondrial transplantation modulates inflammation and apoptosis, alleviating tendinopathy both *in vivo* and *in vitro*. Antioxid (Basel Switzerland). (2021) 10:696. doi: 10.3390/antiox10050696. PMID: 33925007 PMC8146308

[27] Pasquier J Guerrouahen BS Al Thawadi H Ghiabi P Maleki M Abu-Kaoud N . Preferential transfer of mitochondria from endothelial to cancer cells through tunneling nanotubes modulates chemoresistance. J Transl Med. (2013) 11:94. doi: 10.1186/1479-5876-11-94. PMID: 23574623 PMC3668949

[28] Zhang H Yu X Ye J Li H Hu J Tan Y . Systematic investigation of mitochondrial transfer between cancer cells and T cells at single-cell resolution. Cancer Cell. (2023) 41:1788–1802.e10. doi: 10.1016/j.ccell.2023.09.003. PMID: 37816332 PMC10568073

[29] Warburg O Wind F Negelein E . The metabolism of tumors in the body. J Gen Physiol. (1927) 8:519–30. doi: 10.1085/jgp.8.6.519. PMID: 19872213 PMC2140820

[30] Wang Y Patti GJ . The Warburg effect: a signature of mitochondrial overload. Trends Cell Biol. (2023) 33:1014–20. doi: 10.1016/j.tcb.2023.03.013. PMID: 37117116 PMC10600323

[31] Sabharwal SS Schumacker PT . Mitochondrial ROS in cancer: initiators, amplifiers or an Achilles’ heel? Nat Rev Cancer. (2014) 14:709–21. doi: 10.1038/nrc3803. PMID: 25342630 PMC4657553

[32] Kopinski PK Singh LN Zhang S Lott MT Wallace DC . Mitochondrial DNA variation and cancer. Nat Rev Cancer. (2021) 21:431–45. doi: 10.1038/s41568-021-00358-w. PMID: 34045735

[33] Ishikawa K Takenaga K Akimoto M Koshikawa N Yamaguchi A Imanishi H . ROS-generating mitochondrial DNA mutations can regulate tumor cell metastasis. Science. (2008) 320:661–4. doi: 10.1126/science.1156906. PMID: 18388260

[34] Shelton SD House S Martins Nascentes Melo L Ramesh V Chen Z Wei T . Pathogenic mitochondrial DNA mutations inhibit melanoma metastasis. Sci Adv. (2024) 10:eadk8801. doi: 10.1126/sciadv.adk8801. PMID: 39485847 PMC11529715

[35] Park JS Sharma LK Li H Xiang R Holstein D Wu J . A heteroplasmic, not homoplasmic, mitochondrial DNA mutation promotes tumorigenesis via alteration in reactive oxygen species generation and apoptosis. Hum Mol Genet. (2009) 18:1578–89. doi: 10.1093/hmg/ddp069. PMID: 19208652 PMC2733816

[36] van der Windt GJW Everts B Chang C-H Curtis JD Freitas TC Amiel E . Mitochondrial respiratory capacity is a critical regulator of CD8+ T cell memory development. Immunity. (2012) 36:68–78. doi: 10.1016/j.immuni.2011.12.007. PMID: 22206904 PMC3269311

[37] Bengsch B Johnson AL Kurachi M Odorizzi PM Pauken KE Attanasio J . Bioenergetic insufficiencies due to metabolic alterations regulated by the inhibitory receptor PD-1 are an early driver of CD8(+) T cell exhaustion. Immunity. (2016) 45:358–73. doi: 10.1016/j.immuni.2016.07.008. PMID: 27496729 PMC4988919

[38] Scharping NE Menk AV Moreci RS Whetstone RD Dadey RE Watkins SC . The tumor microenvironment represses T cell mitochondrial biogenesis to drive intratumoral T cell metabolic insufficiency and dysfunction. Immunity. (2016) 45:374–88. doi: 10.1016/j.immuni.2016.07.009. PMID: 27496732 PMC5207350

[39] Huang S-C Everts B Ivanova Y O’Sullivan D Nascimento M Smith AM . Cell-intrinsic lysosomal lipolysis is essential for alternative activation of macrophages. Nat Immunol. (2014) 15:846–55. doi: 10.1038/ni.2956. PMID: 25086775 PMC4139419

[40] Colegio OR Chu N-Q Szabo AL Chu T Rhebergen AM Jairam V . Functional polarization of tumour-associated macrophages by tumour-derived lactic acid. Nature. (2014) 513:559–63. doi: 10.1038/nature13490. PMID: 25043024 PMC4301845

[41] Borcherding N Brestoff JR . The power and potential of mitochondria transfer. Nature. (2023) 623:283–91. doi: 10.1038/s41586-023-06537-z. PMID: 37938702 PMC11590279

[42] Saha T Dash C Jayabalan R Khiste S Kulkarni A Kurmi K . Intercellular nanotubes mediate mitochondrial trafficking between cancer and immune cells. Nat Nanotechnol. (2022) 17:98–106. doi: 10.1038/s41565-021-01000-4. PMID: 34795441 PMC10071558

[43] Suh J Lee Y-S . Mitochondria as secretory organelles and therapeutic cargos. Exp Mol Med. (2024) 56:66–85. doi: 10.1038/s12276-023-01141-7. PMID: 38172601 PMC10834547

[44] She Z Xie M Hun M Abdirahman AS Li C Wu F . Immunoregulatory effects of mitochondria transferred by extracellular vesicles. Front Immunol. (2020) 11:628576. doi: 10.3389/fimmu.2020.628576. PMID: 33633746 PMC7900141

[45] Marlein CR Zaitseva L Piddock RE Robinson SD Edwards DR Shafat MS . NADPH oxidase-2 derived superoxide drives mitochondrial transfer from bone marrow stromal cells to leukemic blasts. Blood. (2017) 130:1649–60. doi: 10.1182/blood-2017-03-772939. PMID: 28733324

[46] Ippolito L Morandi A Taddei ML Parri M Comito G Iscaro A . Cancer-associated fibroblasts promote prostate cancer Malignancy via metabolic rewiring and mitochondrial transfer. Oncogene. (2019) 38:5339–55. doi: 10.1038/s41388-019-0805-7. PMID: 30936458

[47] Wang S Liu B Huang J He H Li L Tao A . Cell-in-cell promotes lung cancer Malignancy by enhancing glucose metabolism through mitochondria transfer. Exp Cell Res. (2023) 429:113665. doi: 10.1016/j.yexcr.2023.113665. PMID: 37236579

[48] Kretschmer A Zhang F Somasekharan SP Tse C Leachman L Gleave A . Stress-induced tunneling nanotubes support treatment adaptation in prostate cancer. Sci Rep. (2019) 9:7826. doi: 10.1038/s41598-019-44346-5. PMID: 31127190 PMC6534589

[49] Marlein CR Piddock RE Mistry JJ Zaitseva L Hellmich C Horton RH . CD38-driven mitochondrial trafficking promotes bioenergetic plasticity in multiple myeloma. Cancer Res. (2019) 79:2285–97. doi: 10.1158/0008-5472.CAN-18-0773. PMID: 30622116

[50] Baldwin JG Heuser-Loy C Saha T Schelker RC Slavkovic-Lukic D Strieder N . Intercellular nanotube-mediated mitochondrial transfer enhances T cell metabolic fitness and antitumor efficacy. Cell. (2024) 187:6614–6630.e21. doi: 10.1016/j.cell.2024.08.029. PMID: 39276774 PMC11623344

[51] Lin R-Z Im G-B Luo AC Zhu Y Hong X Neumeyer J . Mitochondrial transfer mediates endothelial cell engraftment through mitophagy. Nature. (2024) 629:660–8. doi: 10.1038/s41586-024-07340-0. PMID: 38693258 PMC11574736

[52] Chen J Fu C-Y Shen G Wang J Xu L Li H . Macrophages induce cardiomyocyte ferroptosis via mitochondrial transfer. Free Radic Biol Med. (2022) 190:1–14. doi: 10.1016/j.freeradbiomed.2022.07.015. PMID: 35933052

[53] Spees JL Olson SD Whitney MJ Prockop DJ . Mitochondrial transfer between cells can rescue aerobic respiration. Proc Natl Acad Sci USA. (2006) 103:1283–8. doi: 10.1073/pnas.0510511103. PMID: 16432190 PMC1345715

[54] Watson DC Bayik D Storevik S Moreino SS Sprowls SA Han J . GAP43-dependent mitochondria transfer from astrocytes enhances glioblastoma tumorigenicity. Nat Cancer. (2023) 4:648–64. doi: 10.1038/s43018-023-00556-5. PMID: 37169842 PMC10212766

[55] Pinto G Saenz-de-Santa-Maria I Chastagner P Perthame E Delmas C Toulas C . Patient-derived glioblastoma stem cells transfer mitochondria through tunneling nanotubes in tumor organoids. Biochem J. (2021) 478:21–39. doi: 10.1042/BCJ20200710. PMID: 33245115 PMC7800365

[56] Marabitti V Vulpis E Nazio F Campello S . Mitochondrial transfer as a strategy for enhancing cancer cell fitness: Current insights and future directions. Pharmacol Res. (2024) 208:107382. doi: 10.1016/j.phrs.2024.107382. PMID: 39218420

[57] He X Zhong L Wang N Zhao B Wang Y Wu X . Gastric cancer actively remodels mechanical microenvironment to promote chemotherapy resistance via MSCs-mediated mitochondrial transfer. Adv Sci (Weinheim Baden-Wurttemberg Ger). (2024) 11:e2404994. doi: 10.1002/advs.202404994. PMID: 39392399 PMC11653701

[58] Civita P M Leite D Pilkington GJ . Pre-clinical drug testing in 2D and 3D human *in vitro* models of glioblastoma incorporating non-neoplastic astrocytes: Tunneling nano tubules and mitochondrial transfer modulates cell behavior and therapeutic respons. Int J Mol Sci. (2019) 20:6017. doi: 10.3390/ijms20236017. PMID: 31795330 PMC6929151

[59] Jing M Xiong X Mao X Song Q Zhang L Ouyang Y . HMGB1 promotes mitochondrial transfer between hepatocellular carcinoma cells through RHOT1 and RAC1 under hypoxia. Cell Death Dis. (2024) 15:155. doi: 10.1038/s41419-024-06536-6. PMID: 38378644 PMC10879213

[60] Goliwas KF Libring S Berestesky E Gholizadeh S Schwager SC Frost AR . Mitochondrial transfer from cancer-associated fibroblasts increases migration in aggressive breast cancer. J Cell Sci. (2023) 136:jcs260419. doi: 10.1242/jcs.260419. PMID: 37358264 PMC10400000

[61] Wang J Liu X Qiu Y Shi Y Cai J Wang B . Cell adhesion-mediated mitochondria transfer contributes to mesenchymal stem cell-induced chemoresistance on T cell acute lymphoblastic leukemia cells. J Hematol Oncol. (2018) 11:11. doi: 10.1186/s13045-018-0554-z. PMID: 29357914 PMC5778754

[62] Porporato PE Filigheddu N Pedro J-S Kroemer G Galluzzi L . Mitochondrial metabolism and cancer. Cell Res. (2018) 28:265–80. doi: 10.1038/cr.2017.155. PMID: 29219147 PMC5835768

[63] Vyas S Zaganjor E Haigis MC . Mitochondria and cancer. Cell. (2016) 166:555–66. doi: 10.1016/j.cell.2016.07.002. PMID: 27471965 PMC5036969

[64] Bajzikova M Kovarova J Coelho AR Boukalova S Oh S Rohlenova K . Reactivation of dihydroorotate dehydrogenase-driven pyrimidine biosynthesis restores tumor growth of respiration-deficient cancer cells. Cell Metab. (2019) 29:399–416.e10. doi: 10.1016/j.cmet.2018.10.014. PMID: 30449682 PMC7484595

[65] Dong L Neuzil J . Targeting mitochondria as an anticancer strategy. Cancer Commun (London England). (2019) 39:63. doi: 10.1186/s40880-019-0412-6. PMID: 31653274 PMC6815053

[66] Weinberg SE Chandel NS . Targeting mitochondria metabolism for cancer therapy. Nat Chem Biol. (2015) 11:9–15. doi: 10.1038/nchembio.1712. PMID: 25517383 PMC4340667

[67] Murphy MP . How mitochondria produce reactive oxygen species. Biochem J. (2009) 417:1–13. doi: 10.1042/BJ20081386. PMID: 19061483 PMC2605959

[68] Zorov DB Juhaszova M Sollott SJ . Mitochondrial reactive oxygen species (ROS) and ROS-induced ROS release. Physiol Rev. (2014) 94:909–50. doi: 10.1152/physrev.00026.2013. PMID: 24987008 PMC4101632

[69] Korolchuk VI Miwa S Carroll B von Zglinicki T . Mitochondria in cell senescence: Is mitophagy the weakest link? EBioMedicine. (2017) 21:7–13. doi: 10.1016/j.ebiom.2017.03.020. PMID: 28330601 PMC5514379

[70] Vats D Mukundan L Odegaard JI Zhang L Smith KL Morel CR . Oxidative metabolism and PGC-1beta attenuate macrophage-mediated inflammation. Cell Metab. (2006) 4:13–24. doi: 10.1016/j.cmet.2006.05.011. PMID: 16814729 PMC1904486

[71] Van den Bossche J O’Neill LA Menon D . Macrophage immunometabolism: Where are we (going)? Trends Immunol. (2017) 38:395–406. doi: 10.1016/j.it.2017.03.001. PMID: 28396078

[72] Hossain F Al-Khami AA Wyczechowska D Hernandez C Zheng L Reiss K . Inhibition of fatty acid oxidation modulates immunosuppressive functions of myeloid-derived suppressor cells and enhances cancer therapies. Cancer Immunol Res. (2015) 3:1236–47. doi: 10.1158/2326-6066.CIR-15-0036. PMID: 26025381 PMC4636942

[73] Michalek RD Gerriets VA Jacobs SR Macintyre AN MacIver NJ Mason EF . Cutting edge: Distinct glycolytic and lipid oxidative metabolic programs are essential for effector and regulatory CD4+ T cell subsets. J Immunol. (2011) 186:3299–303. doi: 10.4049/jimmunol.1003613. PMID: 21317389 PMC3198034

[74] Angelin A Gil-de-Gómez L Dahiya S Jiao J Guo L Levine MH . Foxp3 reprograms T cell metabolism to function in low-glucose, high-lactate environments. Cell Metab. (2017) 25:1282–1293.e7. doi: 10.1016/j.cmet.2016.12.018. PMID: 28416194 PMC5462872

[75] DeNardo DG Ruffell B . Macrophages as regulators of tumour immunity and immunotherapy. Nat Rev Immunol. (2019) 19:369–82. doi: 10.1038/s41577-019-0127-6. PMID: 30718830 PMC7339861

[76] Liu D Gao Y Liu J Huang Y Yin J Feng Y . Intercellular mitochondrial transfer as a means of tissue revitalization. Signal Transduct Target Ther. (2021) 6:65. doi: 10.1038/s41392-020-00440-z. PMID: 33589598 PMC7884415

[77] Guo X Can C Liu W Wei Y Yang X Liu J . Mitochondrial transfer in hematological Malignancies. biomark Res. (2023) 11:89. doi: 10.1186/s40364-023-00529-x. PMID: 37798791 PMC10557299

[78] Mantovani A Marchesi F Malesci A Laghi L Allavena P . Tumour-associated macrophages as treatment targets in oncology. Nat Rev Clin Oncol. (2017) 14:399–416. doi: 10.1038/nrclinonc.2016.217. PMID: 28117416 PMC5480600

[79] Desdín-Micó G Soto-Heredero G Aranda JF Oller J Carrasco E Gabandé-Rodríguez E . T cells with dysfunctional mitochondria induce multimorbidity and premature senescence. Science. (2020) 368:1371–6. doi: 10.1126/science.aax0860. PMID: 32439659 PMC7616968

[80] Yu Y-R Imrichova H Wang H Chao T Xiao Z Gao M . Disturbed mitochondrial dynamics in CD8(+) TILs reinforce T cell exhaustion. Nat Immunol. (2020) 21:1540–51. doi: 10.1038/s41590-020-0793-3. PMID: 33020660

[81] Court AC Le-Gatt A Luz-Crawford P Parra E Aliaga-Tobar V Bátiz LF . Mitochondrial transfer from MSCs to T cells induces Treg differentiation and restricts inflammatory response. EMBO Rep. (2020) 21:e48052. doi: 10.15252/embr.201948052. PMID: 31984629 PMC7001501

[82] Angajala A Lim S Phillips JB Kim J-H Yates C You Z . Diverse roles of mitochondria in immune responses: Novel insights into immuno-metabolism. Front Immunol. (2018) 9:1605. doi: 10.3389/fimmu.2018.01605. PMID: 30050539 PMC6052888

[83] Pang Y Zhang C Gao J . Macrophages as emerging key players in mitochondrial transfers. Front Cell Dev Biol. (2021) 9:747377. doi: 10.3389/fcell.2021.747377. PMID: 34722528 PMC8548694

[84] Davis C-H Marsh-Armstrong N . Discovery and implications of transcellular mitophagy. Autophagy. (2014) 10:2383–4. doi: 10.4161/15548627.2014.981920. PMID: 25484086 PMC4502649

[85] Jackson MV Morrison TJ Doherty DF McAuley DF Matthay MA Kissenpfennig A . Mitochondrial transfer via tunneling nanotubes is an important mechanism by which mesenchymal stem cells enhance macrophage phagocytosis in the *in vitro* and *in vivo* models of ARDS. Stem Cells. (2016) 34:2210–23. doi: 10.1002/stem.2372. PMID: 27059413 PMC4982045

[86] Lu D Jiao X Jiang W Yang L Gong Q Wang X . Mesenchymal stem cells influence monocyte/macrophage phenotype: Regulatory mode and potential clinical applications. BioMed Pharmacother. (2023) 165:115042. doi: 10.1016/j.biopha.2023.115042. PMID: 37379639

[87] Alt EU Senst C Murthy SN Slakey DP Dupin CL Chaffin AE . Aging alters tissue resident mesenchymal stem cell properties. Stem Cell Res. (2012) 8:215–27. doi: 10.1016/j.scr.2011.11.002. PMID: 22265741

[88] Zhang H Menzies KJ Auwerx J . The role of mitochondria in stem cell fate and aging. Development. (2018) 145:dev143420. doi: 10.1242/dev.143420. PMID: 29654217 PMC5964648

[89] Bourebaba L Bourebaba N Galuppo L Marycz K . Artificial mitochondrial transplantation (AMT) reverses aging of mesenchymal stromal cells and improves their immunomodulatory properties in LPS-induced synoviocytes inflammation. Biochim Biophys Acta Mol Cell Res. (2024) 1871:119806. doi: 10.1016/j.bbamcr.2024.119806. PMID: 39098401

[90] Nicolás-Ávila JA Lechuga-Vieco AV Esteban-Martínez L Sánchez-Díaz M Díaz-García E Santiago DJ . A network of macrophages supports mitochondrial homeostasis in the heart. Cell. (2020) 183:94–109.e23. doi: 10.1016/j.cell.2020.08.031. PMID: 32937105

[91] Bai R Cui J . Mitochondrial immune regulation and anti-tumor immunotherapy strategies targeting mitochondria. Cancer Lett. (2023) 564:216223. doi: 10.1016/j.canlet.2023.216223. PMID: 37172686

[92] Wang S Wang J Chen Z Luo J Guo W Sun L . Targeting M2-like tumor-associated macrophages is a potential therapeutic approach to overcome antitumor drug resistance. NPJ Precis Oncol. (2024) 8:31. doi: 10.1038/s41698-024-00522-z. PMID: 38341519 PMC10858952

[93] Chen Y Xiao D Li X . The role of mitochondrial transfer via tunneling nanotubes in the central nervous system: A review. Med (Baltimore). (2024) 103:e37352. doi: 10.1097/MD.0000000000037352. PMID: 38428884 PMC10906627

[94] Barutta F Bellini S Kimura S Hase K Corbetta B Corbelli A . Protective effect of the tunneling nanotube-TNFAIP2/M-sec system on podocyte autophagy in diabetic nephropathy. Autophagy. (2023) 19:505–24. doi: 10.1080/15548627.2022.2080382. PMID: 35659195 PMC9851239

[95] Bian J Zhang D Wang Y Qin H Yang W Cui R . Mitochondrial quality control in hepatocellular carcinoma. Front Oncol. (2021) 11:713721. doi: 10.3389/fonc.2021.713721. PMID: 34589426 PMC8473831

[96] Zhang L Zhang X Liu H Yang C Yu J Zhao W . MTFR2-dependent mitochondrial fission promotes HCC progression. J Transl Med. (2024) 22:73. doi: 10.1186/s12967-023-04845-6. PMID: 38238834 PMC10795309

[97] Huang Q Han Y Shen E Feng Z Peng Y Gao L . MTFR2 shapes a barrier of immune microenvironment in hepatocellular carcinoma. iScience. (2023) 26:105095. doi: 10.1016/j.isci.2022.105095. PMID: 36713263 PMC9881049

[98] Zhang L Zhou B Yang J Ren C Luo J Li Z . MTFR2-mediated fission drives fatty acid and mitochondrial co-transfer from hepatic stellate cells to tumor cells fueling oncogenesis. Adv Sci (Weinheim Baden-Wurttemberg Ger). (2025) 12:e2416419. doi: 10.1002/advs.202416419. PMID: 40365837 PMC12199435

[99] Islam MN Das SR Emin MT Wei M Sun L Westphalen K . Mitochondrial transfer from bone-marrow-derived stromal cells to pulmonary alveoli protects against acute lung injury. Nat Med. (2012) 18:759–65. doi: 10.1038/nm.2736. PMID: 22504485 PMC3727429

[100] Fu H Xie X Zhai L Liu Y Tang Y He S . CX43-mediated mitochondrial transfer maintains stemness of KG-1a leukemia stem cells through metabolic remodeling. Stem Cell Res Ther. (2024) 15:460. doi: 10.1186/s13287-024-04079-3. PMID: 39623456 PMC11613858

[101] Gervasi A D’Aprile S Denaro S Amorini MA Vicario N Parenti R . Connexin 43 role in mitochondrial transfer and homeostasis in the central nervous system. J Cell Physiol. (2025) 240:e70086. doi: 10.1002/jcp.70086. PMID: 40838510 PMC12368938

[102] Qiao X Huang N Meng W Liu Y Li J Li C . Beyond mitochondrial transfer, cell fusion rescues metabolic dysfunction and boosts Malignancy in adenoid cystic carcinoma. Cell Rep. (2024) 43:114652. doi: 10.1016/j.celrep.2024.114652. PMID: 39217612

[103] de Rooij B Polak R Stalpers F Pieters R den Boer ML . Tunneling nanotubes facilitate autophagosome transfer in the leukemic niche. Leukemia. (2017) 31:1651–4. doi: 10.1038/leu.2017.117. PMID: 28400620 PMC5508073

[104] Marcoux G Magron A Sut C Laroche A Laradi S Hamzeh-Cognasse H . Platelet-derived extracellular vesicles convey mitochondrial DAMPs in platelet concentrates and their levels are associated with adverse reactions. Transfusion. (2019) 59:2403–14. doi: 10.1111/trf.15300. PMID: 30973972

[105] Boudreau LH Duchez A-C Cloutier N Soulet D Martin N Bollinger J . Platelets release mitochondria serving as substrate for bactericidal group IIA-secreted phospholipase A2 to promote inflammation. Blood. (2014) 124:2173–83. doi: 10.1182/blood-2014-05-573543. PMID: 25082876 PMC4260364

[106] Mahrouf-Yorgov M Augeul L Da Silva CC Jourdan M Rigolet M Manin S . Mesenchymal stem cells sense mitochondria released from damaged cells as danger signals to activate their rescue properties. Cell Death Differ. (2017) 24:1224–38. doi: 10.1038/cdd.2017.51. PMID: 28524859 PMC5520168

[107] Morrison TJ Jackson MV Cunningham EK Kissenpfennig A McAuley DF O’Kane CM . Mesenchymal stromal cells modulate macrophages in clinically relevant lung injury models by extracellular vesicle mitochondrial transfer. Am J Respir Crit Care Med. (2017) 196:1275–86. doi: 10.1164/rccm.201701-0170OC. PMID: 28598224 PMC5694830

[108] Quintana A Schwindling C Wenning AS Becherer U Rettig J Schwarz EC . T cell activation requires mitochondrial translocation to the immunological synapse. Proc Natl Acad Sci USA. (2007) 104:14418–23. doi: 10.1073/pnas.0703126104. PMID: 17726106 PMC1964825

[109] Suzuki J Umeda M Sims PJ Nagata S . Calcium-dependent phospholipid scrambling by TMEM16F. Nature. (2010) 468:834–8. doi: 10.1038/nature09583. PMID: 21107324

[110] Caicedo A Aponte PM Cabrera F Hidalgo C Khoury M . Artificial mitochondria transfer: Current challenges, advances, and future applications. Stem Cells Int. (2017) 2017:7610414. doi: 10.1155/2017/7610414. PMID: 28751917 PMC5511681

[111] Maeda A Fadeel B . Mitochondria released by cells undergoing TNF-α-induced necroptosis act as danger signals. Cell Death Dis. (2014) 5:e1312. doi: 10.1038/cddis.2014.277. PMID: 24991764 PMC4123071

[112] Narendra DP Jin SM Tanaka A Suen D-F Gautier CA Shen J . PINK1 is selectively stabilized on impaired mitochondria to activate Parkin. PLoS Biol. (2010) 8:e1000298. doi: 10.1371/journal.pbio.1000298. PMID: 20126261 PMC2811155

[113] Bingol B Tea JS Phu L Reichelt M Bakalarski CE Song Q . The mitochondrial deubiquitinase USP30 opposes parkin-mediated mitophagy. Nature. (2014) 510:370–5. doi: 10.1038/nature13418. PMID: 24896179

[114] Chen H Detmer SA Ewald AJ Griffin EE Fraser SE Chan DC . Mitofusins Mfn1 and Mfn2 coordinately regulate mitochondrial fusion and are essential for embryonic development. J Cell Biol. (2003) 160:189–200. doi: 10.1083/jcb.200211046. PMID: 12527753 PMC2172648

[115] Ekstrand MI Falkenberg M Rantanen A Park CB Gaspari M Hultenby K . Mitochondrial transcription factor A regulates mtDNA copy number in mammals. Hum Mol Genet. (2004) 13:935–44. doi: 10.1093/hmg/ddh109. PMID: 15016765

[116] Li B Li B Qiao X Meng W Xie Y Gong J . Targeting mitochondrial transfer as a promising therapeutic strategy. Trends Mol Med. (2025) 31:909–24. doi: 10.1016/j.molmed.2025.04.002. PMID: 40335384

[117] Zhou W Karan KR Gu W Klein H-U Sturm G De Jager PL . Somatic nuclear mitochondrial DNA insertions are prevalent in the human brain and accumulate over time in fibroblasts. PLoS Biol. (2024) 22:e3002723. doi: 10.1371/journal.pbio.3002723. PMID: 39172952 PMC11340991

[118] Scharping NE Menk AV Whetstone RD Zeng X Delgoffe GM . Efficacy of PD-1 blockade is potentiated by metformin-induced reduction of tumor hypoxia. Cancer Immunol Res. (2017) 5:9–16. doi: 10.1158/2326-6066.CIR-16-0103. PMID: 27941003 PMC5340074

[119] Eikawa S Nishida M Mizukami S Yamazaki C Nakayama E Udono H . Immune-mediated antitumor effect by type 2 diabetes drug, metformin. Proc Natl Acad Sci USA. (2015) 112:1809–14. doi: 10.1073/pnas.1417636112. PMID: 25624476 PMC4330733

[120] Akce M Farran B Switchenko JM Rupji M Kang S Khalil L . Phase II trial of nivolumab and metformin in patients with treatment-refractory microsatellite stable metastatic colorectal cancer. J Immunother Cancer. (2023) 11:e007235. doi: 10.1136/jitc-2023-007235. PMID: 37852737 PMC10603338

[121] Burt R Dey A Aref S Aguiar M Akarca A Bailey K . Activated stromal cells transfer mitochondria to rescue acute lymphoblastic leukemia cells from oxidative stress. Blood. (2019) 134:1415–29. doi: 10.1182/blood.2019001398. PMID: 31501154 PMC6856969

[122] Cai J Wang J Huang Y Wu H Xia T Xiao J . ERK/Drp1-dependent mitochondrial fission is involved in the MSC-induced drug resistance of T-cell acute lymphoblastic leukemia cells. Cell Death Dis. (2016) 7:e2459. doi: 10.1038/cddis.2016.370. PMID: 27831567 PMC5260898

[123] Pribila JT Quale AC Mueller KL Shimizu Y . Integrins and T cell-mediated immunity. Annu Rev Immunol. (2004) 22:157–80. doi: 10.1146/annurev.immunol.22.012703.104649. PMID: 15032577

[124] Pinto G Brou C Zurzolo C . Tunneling nanotubes: The fuel of tumor progression? Trends Cancer. (2020) 6:874–88. doi: 10.1016/j.trecan.2020.04.012. PMID: 32471688

[125] Chamoto K Chowdhury PS Kumar A Sonomura K Matsuda F Fagarasan S . Mitochondrial activation chemicals synergize with surface receptor PD-1 blockade for T cell-dependent antitumor activity. Proc Natl Acad Sci USA. (2017) 114:E761–70. doi: 10.1073/pnas.1620433114. PMID: 28096382 PMC5293087

[126] Tanaka K Chamoto K Saeki S Hatae R Ikematsu Y Sakai K . Combination bezafibrate and nivolumab treatment of patients with advanced non-small cell lung cancer. Sci Transl Med. (2022) 14:eabq0021. doi: 10.1126/scitranslmed.abq0021. PMID: 36516270

[127] Al-Habsi M Chamoto K Matsumoto K Nomura N Zhang B Sugiura Y . Spermidine activates mitochondrial trifunctional protein and improves antitumor immunity in mice. Science. (2022) 378:eabj3510. doi: 10.1126/science.abj3510. PMID: 36302005

[128] Macheiner T Fengler VHI Agreiter M Eisenberg T Madeo F Kolb D . Magnetomitotransfer: An efficient way for direct mitochondria transfer into cultured human cells. Sci Rep. (2016) 6:35571. doi: 10.1038/srep35571. PMID: 27767193 PMC5073296

[129] Katayama T Kinugawa S Takada S Furihata T Fukushima A Yokota T . A mitochondrial delivery system using liposome-based nanocarriers that target myoblast cells. Mitochondrion. (2019) 49:66–72. doi: 10.1016/j.mito.2019.07.005. PMID: 31326598

[130] Chang J-C Wu S-L Liu K-H Chen Y-H Chuang C-S Cheng F-C . Allogeneic/xenogeneic transplantation of peptide-labeled mitochondria in Parkinson’s disease: restoration of mitochondria functions and attenuation of 6-hydroxydopamine-induced neurotoxicity. Transl Res. (2016) 170:40–56.e3. doi: 10.1016/j.trsl.2015.12.003. PMID: 26730494

[131] Gäbelein CG Feng Q Sarajlic E Zambelli T Guillaume-Gentil O Kornmann B . Mitochondria transplantation between living cells. PLoS Biol. (2022) 20:e3001576. doi: 10.1371/journal.pbio.3001576. PMID: 35320264 PMC8942278

[132] Maeda H Kami D Maeda R Shikuma A Gojo S . Generation of somatic mitochondrial DNA-replaced cells for mitochondrial dysfunction treatment. Sci Rep. (2021) 11:10897. doi: 10.1038/s41598-021-90316-1. PMID: 34035362 PMC8149667

[133] Headley CA Gautam S Olmo-Fontanez A Garcia-Vilanova A Dwivedi V Akhter A . Extracellular delivery of functional mitochondria rescues the dysfunction of CD4(+) T cells in aging. Adv Sci (Weinheim Baden-Wurttemberg Ger). (2024) 11:e2303664. doi: 10.1002/advs.202303664. PMID: 37990641 PMC10837346

[134] Labarta E de Los Santos MJ Herraiz S Escribá MJ Marzal A Buigues A . Autologous mitochondrial transfer as a complementary technique to intracytoplasmic sperm injection to improve embryo quality in patients undergoing *in vitro* fertilization-a randomized pilot study. Fertil Steril. (2019) 111:86–96. doi: 10.1016/j.fertnstert.2018.09.023. PMID: 30477915

[135] Zhang Q Raoof M Chen Y Sumi Y Sursal T Junger W . Circulating mitochondrial DAMPs cause inflammatory responses to injury. Nature. (2010) 464:104–7. doi: 10.1038/nature08780. PMID: 20203610 PMC2843437

[136] Iyer SS He Q Janczy JR Elliott EI Zhong Z Olivier AK . Mitochondrial cardiolipin is required for Nlrp3 inflammasome activation. Immunity. (2013) 39:311–23. doi: 10.1016/j.immuni.2013.08.001. PMID: 23954133 PMC3779285

[137] Gao Y Mi N Wu W Zhao Y Fan F Liao W . Transfer of inflammatory mitochondria via extracellular vesicles from M1 macrophages induces ferroptosis of pancreatic beta cells in acute pancreatitis. J Extracell Vesicles. (2024) 13:e12410. doi: 10.1002/jev2.12410. PMID: 38320981 PMC10847061

[138] Kiss M Pittet MJ . Mitochondrial control of macrophage polarity in tumors. Immunity. (2025) 58:1618–20. doi: 10.1016/j.immuni.2025.06.008. PMID: 40633525

[139] Chun S An J Kim MS . Mitochondrial transfer between cancer and T cells: Implications for immune evasion. Antioxid (Basel Switzerland). (2025) 14:1008. doi: 10.3390/antiox14081008. PMID: 40867904 PMC12382691

[140] Kuo C-L Ponneri Babuharisankar A Lin Y-C Lien H-W Lo YK Chou H-Y . Mitochondrial oxidative stress in the tumor microenvironment and cancer immunoescape: Foe or friend? J BioMed Sci. (2022) 29:74. doi: 10.1186/s12929-022-00859-2. PMID: 36154922 PMC9511749

